# Sharp Guarantees and Optimal Performance for Inference in Binary and Gaussian-Mixture Models †

**DOI:** 10.3390/e23020178

**Published:** 2021-01-30

**Authors:** Hossein Taheri, Ramtin Pedarsani, Christos Thrampoulidis

**Affiliations:** Department of Electrical and Computer Engineering, University of California, Santa Barbara, CA 93106, USA

**Keywords:** signal processing in machine learning, statistics, optimization

## Abstract

We study convex empirical risk minimization for high-dimensional inference in binary linear classification under both discriminative binary linear models, as well as generative Gaussian-mixture models. Our first result sharply predicts the statistical performance of such estimators in the proportional asymptotic regime under isotropic Gaussian features. Importantly, the predictions hold for a wide class of convex loss functions, which we exploit to prove bounds on the best achievable performance. Notably, we show that the proposed bounds are tight for popular binary models (such as signed and logistic) and for the Gaussian-mixture model by constructing appropriate loss functions that achieve it. Our numerical simulations suggest that the theory is accurate even for relatively small problem dimensions and that it enjoys a certain universality property.

## 1. Introduction

### 1.1. Motivation

Classical estimation theory studies problems in which the number of unknown parameters *n* is small compared to the number of observations *m*. In contrast, modern inference problems are typically *high-dimensional*, that is *n* can be of the same order as *m*. Examples are abundant in a wide range of signal processing and machine learning applications such as medical imaging, wireless communications, recommendation systems, etc. Classical tools and theories are not applicable in these modern inference problems [1]. As such, over the last two decades or so, the study of high-dimensional estimation problems has received significant attention.

Perhaps the most well-studied setting is that of noisy linear observations (namely, linear regression). The literature on the topic is vast with remarkable contributions from the statistics, signal processing and machine learning communities. Several recent works focus on the *proportional/linear asymptotic regime* and derive *sharp* results on the inference performance of appropriate convex optimization methods (e.g., [2,3,4,5,6,7,8,9,10,11,12,13,14,15,16,17,18,19,20,21,22,23]). These works show that, albeit challenging, *sharp* results are advantageous over loose order-wise bounds. Not only do they allow for accurate comparisons between different choices of the optimization parameters, but they also form the basis for establishing optimal such choices as well as fundamental performance limitations (e.g., [12,14,15,16,24,25,26]).

This paper takes this recent line of work a step further by demonstrating that results of this nature can be achieved in binary observation models. While we depart from the previously studied linear regression model, we remain faithful to the requirement and promise of sharp results. Binary models are popularly applicable in a wide range of signal-processing (e.g., highly quantized measurements) and machine learning (e.g., binary classification) problems. We derive sharp asymptotics for a rich class of convex optimization estimators, which include least-squares, logistic regression and hinge loss as special cases. Perhaps more interestingly, we use these results to derive fundamental performance limitations and design optimal loss functions that provably outperform existing choices. Our results hold both for discriminative and generative data models.

In Section 1.2, we formally introduce the problem setup. The paper’s main contributions and organization are presented in Section 1.4. A detailed discussion of prior art follows in Section 1.5.

**Notation** **1.** The symbols P(·), $E\left[\cdot \right]$ and Var[·] denote probability, expectation and variance, respectively. We use boldface notation for vectors. ${∥v∥}_{2}$ denotes the Euclidean norm of a vector v. We write i∈[m] for i=1,2,…,m. When writing $x_{*}=\arg\min_{x}f\left(x\right),$ we let the operator argmin return any one of the possible minimizers of f. For all x∈R, Φ(x) is the cumulative distribution function of standard normal and Gaussian Q-function at x is defined as Q(x)=1−Φ(x).

### 1.2. Data Models

Consider *m* data pairs ${\left(y_{i},a_{i}\right)}_{i=1}^{m}$ generated i.i.d from one of the following two models such that $y_{i}\in \left\{-1,+1\right\}$ and $a_{i}\in R^{n}$ for all i∈[m].

**Binary models with Gaussian features**: Here, the feature/measurement vectors $a_{i},i\in \left[n\right]$ have i.i.d Gaussian entries, i.e., $a_{i}∼N\left(0,I_{n}\right).$ Given the feature vector $a_{i}$, the corresponding label takes the form

(1) $$\begin{array}{c} y_{i}=f\left(a_{i}^{T}x_{0}\right),\;\;i\in \left[m\right], \end{array}$$

for some unknown true signal $x_{0}\in R^{n}$ and a label/link function f:R→{−1,+1} a (possibly random) binary function. Some popular examples for the label function *f* include the following:

- *(Noisy) Signed*: $\left\{\begin{array}{cc} \mathrm{sign}(a_{i}^{T}x_{0}) & ,\;w.p.\;1-\varepsilon ,\\ -\mathrm{sign}(a_{i}^{T}x_{0}) & ,\;w.p.\;\varepsilon , \end{array}\right.\;\;\;\mathrm{where}\;\varepsilon \in \left[0,1/2\right].$
- *Logistic*: $y_{i}=\left\{\begin{array}{cc} +1 & ,\;w.p.\;\frac{1}{1+\exp(-a_{i}^{T}x_{0})},\\ -1 & ,\;w.p.\;1-\frac{1}{1+\exp(-a_{i}^{T}x_{0})}. \end{array}\right.$
- *Probit*: $y_{i}=\left\{\begin{array}{cc} +1 & ,\;w.p.\;\Phi (a_{i}^{T}x_{0}),\\ -1 & ,\;w.p.\;1-\Phi (a_{i}^{T}x_{0}). \end{array}\right.$

We remark that when the signal strength $∥x_{0}{∥}_{2}\to +\infty$, logistic and Probit label functions approach the signed model (i.e., noisy-signed function with ε=0).

Throughout, we assume that $∥x_{0}{∥}_{2}=1$. This assumption is without loss of generality since the norm of $x_{0}$ can always be absorbed in the link function. Indeed, letting $∥x_{0}{∥}_{2}=r$, we can always write the measurements as $f\left(a^{T}x_{0}\right)=\tilde{f}\left(a^{T}{\tilde{x}}_{0}\right)$, where ${\tilde{x}}_{0}=x_{0}/r$ (hence, $∥{\tilde{x}}_{0}{∥}_{2}=1$) and $\tilde{f}\left(t\right)=f\left(rt\right)$. We make no further assumptions on the distribution of the true vector $x_{0}$.

**Gaussian-mixture model**: In Section 5, we also study the following generative Gaussian-mixture model (GMM):

(2) $$\begin{array}{c} y_{i}=\left\{\begin{array}{cc} +1 & ,\;w.p.\;\pi ,\\ -1 & ,\;w.p.\;1-\pi , \end{array}\right.\;,\;a_{i}|y_{i}∼N\left(y_{i}x_{0},I_{n}\right),\;i\in \left[m\right]. \end{array}$$

Above, π∈[0,1] is the prior of class +1 and $x_{0}\in R^{n}$ is the true signal, which here represents the mean of the features.

### 1.3. Empirical Risk Minimization

We study the performance of *empirical-risk minimization (ERM)* estimators ${\hat{x}}_{\ell }$ of $x_{0}$ that solve the following optimization problem for some *convex* loss function ℓ:R→R

(3) $$\begin{array}{c} {\hat{x}}_{\ell }:=\arg\min_{x}\frac{1}{m}\sum_{i=1}^{m}\ell \left(y_{i}a_{i}^{T}x\right). \end{array}$$

**Loss function.** Different choices for *ℓ* lead to popular specific estimators including the following:

- *Least Squares (LS):*$\ell \left(t\right)={\left(t-1\right)}^{2}$,
- *Least-Absolute Deviations (LAD):*ℓ(t)=|t−1|,
- *Logistic Loss:*ℓ(t)=log(1+exp(−t)),
- *Exponential Loss:*ℓ(t)=exp(−t),
- *Hinge Loss:*ℓ(t)=max{1−t,0}.

**Performance Measure.** We measure performance of the estimator ${\hat{x}}_{\ell }$ by the value of its correlation to $x_{0}$, i.e.,

(4) $$\begin{array}{c} \mathrm{corr}\left(\;{\hat{x}}_{\ell }\;;\;x_{0}\;\right):=\frac{\;\langle {\hat{x}}_{\ell },x_{0}\rangle \;}{∥{\hat{x}}_{\ell }{∥}_{2}{∥x_{0}∥}_{2}}\in \left[-1,1\right]. \end{array}$$

Obviously, we seek estimates that maximize correlation. While correlation is the measure of primal interest, our results extend rather naturally to other prediction metrics, such as classification error given by (e.g., see [27] (Section D.2.)),

(5) $$\begin{array}{c} E_{\ell }:=E_{a,y}\left[1_{\left\{y\neq \;\mathrm{sign}\left(\langle {\hat{x}}_{\ell },\;a\rangle \right)\right\}}\right]. \end{array}$$

Expectation in (5) is derived based on a test sample (a,y) from the same distribution of the training set.

### 1.4. Contributions and Organization

As mentioned, our techniques naturally apply to both binary Gaussian and Gaussian-mixture models. For concreteness, we focus our presentation on the former models (see Section 2, Section 3 and Section 4.1). Then, we extend our results to Gaussian mixtures in Section 5. Numerical simulations corroborating our theoretical findings for both models are presented in Section 6.

Now, we state the paper’s main contributions:

- **Precise Asymptotics**: We show that the absolute value of correlation of ${\hat{x}}_{\ell }$ to the true vector $x_{0}$ is sharply predicted by $\sqrt{1/(1+\sigma_{\ell }^{2})}$ where the “effective noise” parameter $\sigma_{\ell }$ can be explicitly computed by solving a system of three non-linear equations in three unknowns. We find that the system of equations (and, thus, the value of $\sigma_{\ell }$) depends on the loss function *ℓ* through its Moreau envelope function. Our prediction holds in the linear asymptotic regime in which m,n→∞ and m/n→δ>1 (see Section 2).
- **Fundamental Limits**: We establish fundamental limits on the performance of convex optimization-based estimators by computing an upper bound on the best possible correlation performance among all convex loss functions. We compute the upper bound by solving a certain nonlinear equation and we show that such a solution exists for all δ>1 (see Section 3.1).
- **Optimal Performance and (sub)-optimality of LS for binary models**: For certain binary models including signed and logistic, we find the loss functions that achieve the optimal performance, i.e., they attain the previously derived upper bound (see Section 3.2). Interestingly, for logistic and Probit models with $∥x_{0}{∥}_{2}=1$, we prove that the correlation performance of least-squares (LS) is at least as good 0.9972 and 0.9804 times the optimal performance. However, as $∥x_{0}{∥}_{2}$ grows large, logistic and Probit models approach the signed model, in which case LS becomes sub-optimal (see Section 4.1).
- **Extension to the Gaussian-Mixture Model**: In Section 5, we extend the fundamental limits and the system of equations to the Gaussian-mixture model. Interestingly, our results indicate that, for this model, LS is optimal among all convex loss functions for all δ>1.
- **Numerical Simulations**: We do numerous experiments to specialize our results to popular models and loss functions, for which we provide simulation results that demonstrate the accuracy of the theoretical predictions (see Section 6 and Appendix E).

**Figure 1:**
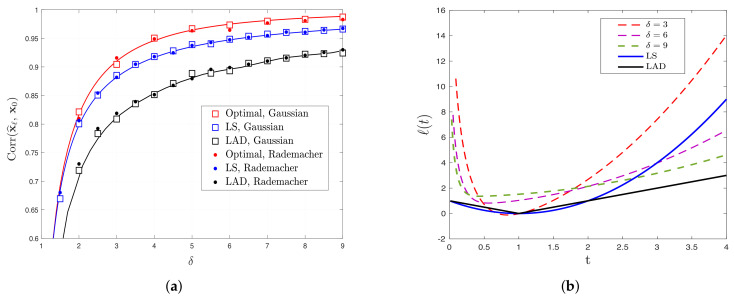
(**a**) Comparison between theoretical (solid lines) and empirical (markers) performance for least-squares (LS) and least-absolute deviations (LAD), as predicted by Theorem 1, and the optimal performance, as predicted by the upper bound of Theorem 2, for the signed model. The squares and circles denote the empirical performance for Gaussian and Rademacher features, respectively. (**b**) Illustrations of optimal loss functions for the signed model for different values of δ according to Theorem 3.

Figure 1 contains a pictorial preview of our results described above for the special case of signed measurements. First, Figure 1a depicts the correlation performance of LS and LAD estimators as a function of the aspect ratio δ. Both theoretical predictions and numerical results are shown; note the close match between theory and empirical results for both i.i.d. Gaussian (shown by circles) and i.i.d. Rademacher (shown by squares) distributions of the feature vectors for even small dimensions. Second, the red line on the same figure shows the upper bound derived in this paper—there is no convex loss function that results in correlation exceeding this line. Third, we show that the upper bound can be achieved by the loss functions depicted in Figure 1b for several values of δ. We solve (3) for this choice of loss functions using gradient descent and numerically evaluate the achieved correlation performance. The recorded values are compared in Table 1 to the corresponding values of the upper bound; again, note the close agreement between the values as predicted by the findings of this paper, which suggests that the fundamental limits derived in this paper hold for sub-Gaussian features. We present corresponding results for the logistic and Probit models in Section 6 and for the noisy-signed model in Appendix E.

**Figure 2:**
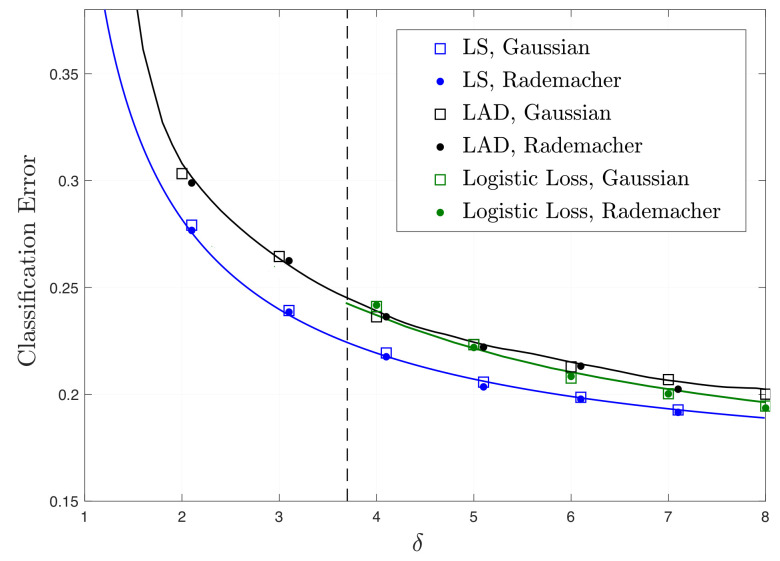
Theoretical (solid lines) and empirical (markers) results of classification risk in GMM as in Theorem 4 and (39) for LS, LAD and logistic loss functions as a function of δ for r=1. The vertical line represents the threshold $\delta^{☆}\approx 3.7$ as evaluated by (36). Logistic loss gives unbounded solution if and only if $\delta <\delta^{☆}$.

**A remark on the Gaussianity assumption.** Our results on precise asymptotics (to which our study of fundamental limits rely upon) hold rigorously for the two data models in Section 1.2, in which the feature vectors have entries i.i.d. standard Gaussian. However, we conjecture that the Gaussianity assumption can be relaxed. As partial numerical evidence, note in Figure 1a the perfect match of our theory with the empirical performance over data in which the feature vectors $a_{i},i\in \left[m\right]$ have entries i.i.d. Rademacher (i.e., centered Bernoulli with probability 1/2). Figure 2 shows corresponding results for the Gaussian-mixture model. Our conjecture that the so-called *universality* property holds in our setting is also in line with similar numerical observations and partial theoretical evidence previously made for linear regression settings [7,28,29,30,31]. A formal proof of universality of our results is beyond the scope of this paper. However, we remark that, as long as the asymptotic predictions of Section 2 enjoy this property, then all our results on fundamental performance limits and optimal functions automatically hold under the same relaxed assumptions.

### 1.5. Related Works

Over the past two decades, there has been a long list of works that derive statistical guarantees for high-dimensional estimation problems. Many of these are concerned with convex optimization-based inference methods. Our work is most closely related to the following three lines of research.

(a) Sharp asymptotics for linear measurements.

Most of the results in the literature of high-dimensional statistics are order-wise in nature. Sharp asymptotic predictions have only more recently appeared in the literature for the case of noisy linear measurements with Gaussian measurement vectors. There are by now three different approaches that have been used towards asymptotic analysis of convex regularized estimators: (i) the one that is based on the approximate message passing (AMP) algorithm and its state-evolution analysis (e.g., [5,8,14,20,32,33,34]); (ii) the one that is based on Gaussian process (GP) inequalities, specifically on the convex Gaussian min-max Theorem (CGMT) (e.g., [9,10,13,15,18,19]); and (iii) the “leave-one-out” approach [11,35]. The three approaches are quite different to each other and each comes with its unique distinguishing features and disadvantages. A detailed comparison is beyond our scope.

Our results in Theorems 2 and 3 for achieving the best performance across all loss functions is complementary to [12] (Theorem 1) and the work of Advani and Ganguli [16], who proposed a method for deriving optimal loss function and measuring its performance, albeit for *linear* models. Instead, we study binary models. The optimality of regularization for linear measurements is recently studied in [22].

In terms of analysis, we follow the GP approach and build upon the CGMT. Since the previous works are concerned with linear measurements, they consider estimators that solve minimization problems of the form

(6) $$\begin{array}{c} \hat{x}:=\arg\min_{x}\sum_{i=1}^{m}\tilde{\ell }\left(y_{i}-a_{i}^{T}x\right)+rR\left(x\right) \end{array}$$

Specifically, the loss function $\tilde{\ell }$ penalizes the residual. In this paper, we show that the CGMT is applicable to optimization problems in the form of (3). For our case of binary observations, (3) is more general than (6). To see this, note that, for $y_{i}\in \pm 1$ and popular symmetric loss functions $\tilde{\ell }\left(t\right)=\tilde{\ell }\left(-t\right)$, e.g., least-squares (LS), (3) results in (6) by choosing $\ell \left(t\right)=\tilde{\ell }\left(t-1\right)$ in the former. Moreover, (3) includes several other popular loss functions such as the logistic loss and the hinge loss which cannot be expressed by (6).

(b) One-bit compressed sensing.

Our work naturally relates to the literature on one-bit compressed sensing (CS) [36]. The vast majority of performance guarantees for one-bit CS are order-wise in nature (e.g., [37,38,39,40,41,42]). To the best of our knowledge, the only existing sharp results are presented in [43] for Gaussian measurement vectors, which studies the asymptotic performance of regularized LS. Our work can be seen as a direct extension of the work in [43] to loss functions beyond least-squares (see Section 4.1 for details).

Similar to the generality of our paper, Genzel [41] also studied the high-dimensional performance of general loss functions. However, in contrast to our results, their performance bounds are loose (order-wise); as such, they are not informative about the question of optimal performance which we also address here.

(c) Classification in high-dimensions.

In [44,45], the authors studied the high-dimensional performance of maximum-likelihood (ML) estimation for the logistic model. The ML estimator is a special case of (3) and we consider general binary models. In addition, their analysis is based on the AMP framework. The asymptotics of logistic loss under different classification models is also recently studied in [46]. In yet another closely related recent work [47], the authors extended the results of Sur and Candes [45] to regularized ML by using the CGMT. Instead, we present results for general convex loss functions and for binary linear models. Importantly, we also study performance bounds and optimal loss functions.

**Figure 3:**
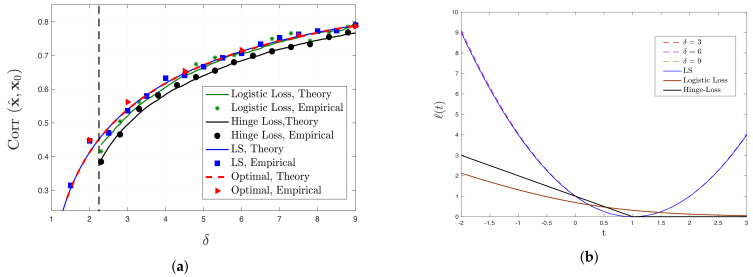
(**a**) Comparison between analytical and empirical results for the performance of LS, logistic loss, hinge loss and optimal loss function for logistic model. The vertical dashed line represents $\delta_{f}^{☆}\approx 2.275$, as evaluated by (35). (**b**) Illustrations of optimal loss functions for different values of δ, derived according to Theorem 3 for logistic model. To signify the similarity of optimal loss function to the LS loss, the optimal loss functions (hardly visible) are scaled such that ℓ(1)=0 and ℓ(2)=1.

We also remark on the following closely related parallel works. While the conference version of this paper was being reviewed, the CGMT was applied by Montanari et al. [48] and Deng et al. [49] to determine the generalization performance of max-margin linear classifiers in a binary classification setting. In essence, these results are complementary to the results of our paper in the following sense. Consider a binary classification setting under the logistic model and Gaussian regressors. As discussed in Section 4.2, the optimal set of (3) is bounded with probability approaching one if and only if $\delta >\delta_{f}^{☆}$, for appropriate threshold $\delta_{f}^{☆}$ determined for first time in [44] (see also Figure 3a). Our results hold in this regime. In contrast, the papers by Montanari et al. [48] and Deng et al. [49] study the regime $\delta <\delta_{f}^{☆}$.

We close this section by mentioning works that build on our results and appeared after the initial submission of this paper. The paper by Mignacco et al. [50] studies sharp asymptotics of ridge-regularized ERM with an intercept for Gaussian-mixture models. In [27], we extend the results of this paper on fundamental limits and optimality to the case of ridge-regularized ERM (see also the concurrent work by Aubin et al. [51]).

## 2. Sharp Performance Guarantees

### 2.1. Definitions

**Moreau Envelopes.** Before stating the first result, we need a definition. We write

$$M_{\ell }\left(x;\lambda \right):=\min_{v}\frac{1}{2\lambda }{\left(x-v\right)}^{2}+\ell \left(v\right),$$

for the *Moreau envelope function* of the loss ℓ:R→R at *x* with parameter λ>0. The minimizer (which is unique by strong convexity) is known as the *proximal operator* of *ℓ* at *x* with parameter λ and we denote it as ${\mathrm{prox}}_{\ell }\left(x;\lambda \right)$. A useful property of the Moreau envelope function is that it is continuously differentiable with respect to both *x* and λ [52]. We denote these derivatives as follows

$$\begin{array}{cc} M_{\ell ,1}^{\prime }\left(x;\lambda \right) & :=\frac{\partial M_{\ell }\left(x;\lambda \right)}{\partial x},\\ M_{\ell ,2}^{\prime }\left(x;\lambda \right) & :=\frac{\partial M_{\ell }\left(x;\lambda \right)}{\partial \lambda }. \end{array}$$

### 2.2. A System of Equations

As we show shortly the asymptotic performance of the optimization in (3) is tightly connected to the solution of a certain system of nonlinear equations, which we introduce here. Specifically, define random variables G,S and *Y* as follows:

(7) $$\begin{array}{c} G,S\overset{i.i.d.}{∼}N\left(0,1\right)\;\mathrm{and}\;Y=f\left(S\right), \end{array}$$

and consider the following system of non-linear equations in three unknowns (μ,α≥0,λ≥0):

(8a) $$\begin{array}{cc} E\left[Y\;S\cdot M_{\ell ,1}^{\prime }\left(\alpha G+\mu SY;\lambda \right)\right] & =0, \end{array}$$

(8b) $$\begin{array}{cc} \lambda^{2}\;\delta \;E\left[\;{\left(M_{\ell ,1}^{\prime }\left(\alpha G+\mu SY;\lambda \right)\right)}^{2}\;\right] & =\alpha^{2}, \end{array}$$

(8c) $$\begin{array}{cc} \lambda \;\delta \;E\left[G\cdot M_{\ell ,1}^{\prime }\left(\alpha G+\mu SY;\lambda \right)\right] & =\alpha . \end{array}$$

The expectations are with respect to the randomness of the random variables *G*, *S* and *Y*. We remark that the equations are well defined even if the loss function *ℓ* is not differentiable. In Appendix A, we summarize some well-known properties of the Moreau envelope function and use them to simplify (8) for differentiable loss functions.

### 2.3. Asymptotic Prediction

We are now ready to state our first main result.

**Theorem** **1.** (Sharp Asymptotics). *Assume data generated from the binary model with Gaussian features and assume δ>1 such that the set of minimizers in (3) is bounded and the system of Equation (8) has a unique solution (μ,α≥0,λ≥0), such that μ≠0. Let ${\hat{x}}_{\ell }$ be as in (3). Then, in the limit of m,n→+∞, m/n→δ, it holds with probability one that*

(9) $$\begin{array}{c} \lim_{n\to \infty }\mathrm{corr}\left(\;{\hat{x}}_{\ell }\;;\;x_{0}\;\right)=\frac{\mu }{\sqrt{\mu^{2}+\alpha^{2}}}. \end{array}$$

*Moreover,*

(10) $$\begin{array}{c} \lim_{n\to \infty }{∥{\hat{x}}_{\ell }-\mu \cdot \frac{x_{0}}{∥x_{0}{∥}_{2}}∥}_{2}^{2}=\alpha^{2}. \end{array}$$

**Figure 4:**
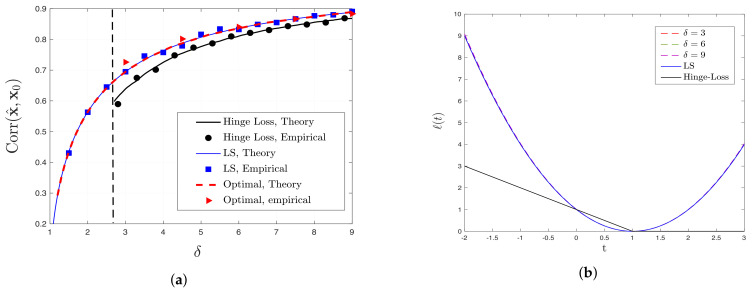
(**a**) Comparison between analytical and empirical results for the performance of LS, hinge loss and optimal loss function for Probit model. The vertical dashed line represents $\delta_{f}^{☆}\approx 2.699$, as evaluated by (35). (**b**) Illustrations of optimal loss functions for different values of δ derived according to Theorem 3 for Probit model. To signify the similarity of optimal loss function to the LS loss, the optimal loss functions (hardly visible) are scaled such that ℓ(1)=0 and ℓ(2)=1.

Theorem 1 holds for any convex loss function. In Section 4, we specialize the result to specific popular choices and also present numerical simulations that confirm the validity of the predictions (see Figure 1a, Figure 3a, Figure 4a and Figure A4a,b). Before that, we include a few remarks on the conditions, interpretation and implications of the theorem. The proof is deferred to Appendix B and uses the convex Gaussian min-max theorem (CGMT) [13,15].

**Remark** **1.** (The Role of μ and α). *According to (9), the prediction for the limiting behavior of the correlation value is given in terms of an effective noise parameter $\sigma_{\ell }:=\alpha /\mu$, where μ and α are unique solutions of (8). The smaller is the value of $\sigma_{\ell }$ is, the larger the correlation value becomes. While the correlation value is fully determined by the ratio of α and μ, their individual role is clarified in (10). Specifically, according to (10), ${\hat{x}}_{\ell }$ is a biased estimate of the true $x_{0}$ and μ represents exactly the correlation bias term. In other words, solving (3) returns an estimator that is close to a μ-scaled version of $x_{0}$. When $x_{0}$ and ${\hat{x}}_{\ell }$ are scaled appropriately, the $\ell_{2}$-norm of their difference converges to α*.

**Remark** **2.** (Why δ>1). *The theorem requires that δ>1 (equivalently, m>n asymptotically). Here, we show that this condition is necessary for Equations (8) to have a bounded solution. To see this, take squares in both sides of (8c) and divide by (8b) to find that*

$$\delta =\frac{E\left[\;{\left(M_{\ell ,1}^{\prime }\left(\alpha G+\mu SY;\lambda \right)\right)}^{2}\;\right]}{{\left(E\left[G\cdot M_{\ell ,1}^{\prime }\left(\alpha G+\mu SY;\lambda \right)\right]\right)}^{2}}\geq 1.$$

*The inequality follows by applying Cauchy–Schwarz and using the fact that $E[G^{2}]=1$.*

**Remark** **3.** (On the Existence of a Solution to (8)). *While δ>1 is a necessary condition for the equations in (8) to have a solution, it is not sufficient in general. This depends on the specific choice of the loss function. For example, in Section 4.1, we show that, for the squared loss $\ell \left(t\right)={\left(t-1\right)}^{2}$, the equations have a unique solution iff δ>1. On the other hand, for logistic loss and hinge loss, it is argued in Section 4.2 that there exists a threshold value $\delta_{f}^{☆}>2$ such that the set of minimizers in (3) is unbounded if $\delta <\delta_{f}^{☆}$. In this case, the assumptions of Theorem 1 do not hold. We conjecture that, for these choices of loss, Equations (8) are solvable iff $\delta >\delta_{f}^{☆}$. Justifying this conjecture and further studying more general sufficient and necessary conditions under which the Equation (8) admit a solution is left to future work. However, in what follows, given such a solution, we prove that it is unique for a wide class of convex loss functions of interest.*

**Remark** **4.** (On the Uniqueness of Solutions to (8)). *We show that, if the system of equations in (8) has a solution, then it is unique provided that ℓ is strictly convex, continuously differentiable and its derivative satisfies $\ell^{\prime }\left(0\right)\neq 0$. For instance, this class includes the square, the logistic and the exponential losses. However, it excludes non-differentiable functions such as the LAD and hinge loss. We believe that the differentiability assumption can be relaxed without major modification in our proof, but we leave this for future work. Our result is summarized in Proposition 1 below.*

**Proposition** **1.** (Uniqueness). *Assume that the loss function ℓ:R→R has the following properties: (i) it is proper strictly convex; and (ii) it is continuously differentiable and its derivative $\ell^{\prime }$ is such that $\ell^{\prime }\left(0\right)\neq 0$. Further, assume that the (possibly random) link function f is such that SY=Sf(S),S∼N(0,1) has strictly positive density on the real line. The following statement is true. For any δ>1, if the system of equations in (8) has a bounded solution, then it is unique.*

*The detailed proof of Proposition 1 is deferred to Appendix B.5. Here, we highlight some key ideas. The CGMT relates—in a rather natural way—the original ERM optimization (3) to the following deterministic min-max optimization on four variables*

(11) $$\begin{array}{c} \min_{\alpha >0,\mu ,\tau >0}\max_{\gamma >0}F\left(\alpha ,\mu ,\tau ,\gamma \right):=\frac{\gamma \tau }{2}-\frac{\alpha \gamma }{\sqrt{\delta }}+E\left[M_{\ell }\left(\alpha G+\mu YS;\frac{\tau }{\gamma }\right)\right]. \end{array}$$

*In Appendix B.4, we show that the optimization above is convex-concave for any lower semi-continuous, proper and convex function ℓ:R→R. Moreover, it is shown that one arrives at the system of equations in (8) by simplifying the first-order optimality conditions of the min-max optimization in (11). This connection is key to the proof of Proposition 1. Indeed, we prove uniqueness of the solution (if such a solution exists) to (8), by proving instead that the function F(α,μ,τ,γ) above is (jointly) strictly convex in (α,μ,τ) and strictly concave in γ, provided that ℓ satisfies the conditions of the proposition. Next, let us briefly discuss how strict convex-concavity of (11) can be shown. For concreteness, we only discuss strict convexity here; the ideas are similar for strict concavity. At the heart of the proof of strict convexity of F is understanding the properties of the expected Moreau envelope function $\Omega :R_{+}\times R\times R_{+}\times R_{+}\to R$ defined as follows:*

$$\Omega \left(\alpha ,\mu ,\tau ,\gamma \right):=E\left[M_{\ell }\left(\alpha G+\mu YS;\frac{\tau }{\gamma }\right)\right].$$

*Specifically, we prove in Proposition A7 in Appendix A.6 that if ℓ is strictly convex, differentiable and does not attain its minimum at 0, then Ω is strictly convex in (α,μ,τ) and strictly concave in γ. It is worth noting that the Moreau envelope function $M_{\ell }\left(\alpha g+\mu ys;\tau \right)$ for fixed g,s and y=f(s) is not necessarily strictly convex. Interestingly, we show that the expected Moreau envelope has this desired feature. We refer the reader to Appendix A.6 and Appendix B.5 for more details.*

## 3. On Optimal Performance

### 3.1. Fundamental Limits

In this section, we establish fundamental limits on the performance of (3) by deriving an upper bound on the absolute value of correlation $\mathrm{corr}\left(\;{\hat{x}}_{\ell }\;;\;x_{0}\;\right)$ that holds for *all* choices of loss functions satisfying Theorem 1. The result builds on the prediction of Theorem 1. In view of (9), upper bounding correlation is equivalent to lower bounding the effective noise parameter $\sigma_{\ell }=\alpha /\mu$. Theorem 2 derives such a lower bound.

Before stating the theorem, we need a definition. For a random variable *H* with density $p_{H}\left(h\right)$ that has a derivative $p_{H}^{\prime }\left(h\right),\forall h\in R$, we denote its score function $\xi_{H}\left(h\right):=\frac{\partial }{\partial h}\log p_{H}\left(h\right)=\frac{p_{H}^{\prime }\left(h\right)}{p_{H}\left(h\right)}$. Then, the Fisher information of *H*, denoted by $I\left(H\right)\in R_{+}$, is defined as follows (e.g., [53] (Sec. 2)):

$$I\left(H\right):=E\left[\;{\left(\xi_{H}\left(H\right)\right)}^{2}\;\right].$$

**Theorem** **2.** (Best Achievable Performance). *Let the assumptions and notation of Theorem 1 hold and recall the definition of random variables G,S and Y in (7). For σ>0, define a new random variable $W_{\sigma }:=\sigma G+SY,$ and the function κ:(0,∞]→[0,1] as follows,*

$$\begin{array}{c} \kappa \left(\sigma \right):=\frac{\sigma^{2}\left(\sigma^{2}I\left(W_{\sigma }\right)+I\left(W_{\sigma }\right)-1\right)}{1+\sigma^{2}\left(\sigma^{2}I\left(W_{\sigma }\right)-1\right)}. \end{array}$$

*Further, define $\sigma_{\mathrm{opt}}$ as follows,*

(12) $$\begin{array}{c} \sigma_{\mathrm{opt}}:=\min\left\{\sigma \geq 0:\kappa \left(\sigma \right)=\frac{1}{\delta }\right\}. \end{array}$$

*Then, for $\sigma_{\ell }:=\frac{\alpha }{\mu }$, it holds that $\sigma_{\ell }\geq \sigma_{\mathrm{opt}}$.*

The theorem above establishes an upper bound on the best possible correlation performance among all convex loss functions. In Section 3.2, we show that this bound is often tight, i.e., there exists a loss function that achieves the specified best possible performance.

**Remark** **5.** *Theorem 2 complements the results in [12,14] (Lem. 3.4) and [15] (Rem. 5.3.3), in which the authors considered only linear regression. In particular, Theorem 2 shows that it is possible to achieve results of this nature for the more challenging setting of binary classification considered here.*

**Proof** **of** **Theorem** **2.** Fix a loss function *ℓ* and let (μ≠0,α>0,λ≥0) be a solution to (8), which by assumptions of Theorem 1 is unique. The first important observation is that the error of a loss function is unique up to a multiplicative constant. To see this, consider an arbitrary loss function ℓ(t) and let ${\hat{x}}_{\ell }$ be a minimizer in (3). Now, consider (3) with the following loss function instead, for some arbitrary constants $C_{1}>0,C_{2}\neq 0$:

(13) $$\begin{array}{c} \hat{\ell }\left(t\right):=\frac{1}{C_{1}}\ell \left(C_{2}t\right). \end{array}$$

It is not hard to see that $\frac{1}{C_{2}}\;{\hat{x}}_{\ell }$ is the minimizer for $\hat{\ell }$. Clearly, $\frac{1}{C_{2}}{\hat{x}}_{\ell }$ has the same correlation value with $x_{0}$ as ${\hat{x}}_{\ell }$, showing that the two loss functions *ℓ* and $\hat{\ell }$ perform the same. With this observation in mind, consider the function $\hat{\ell }:R\to R$ such that $\hat{\ell }\left(t\right)=\frac{\lambda }{\mu^{2}}\ell \left(\mu \;t\right)$. Then, notice that

$$M_{\ell ,1}^{\prime }\left(x;\lambda \right)=\frac{1}{\lambda }M_{\hat{\ell },1}^{\prime }\left(x/\mu ;1\right).$$

Using this relation in (8) and setting $\sigma :=\sigma_{\ell }=\alpha /\mu$, the system of equations in (8) can be equivalently rewritten in the following convenient form,

(14a) $$\begin{array}{cc} & E\left[Y\;S\cdot M_{\hat{\ell },1}^{\prime }\left(W_{\sigma };1\right)\right]=0, \end{array}$$

(14b) $$\begin{array}{cc} & E\left[\;{\left(M_{\hat{\ell },1}^{\prime }\left(W_{\sigma };1\right)\right)}^{2}\;\right]=\sigma^{2}/\delta \;, \end{array}$$

(14c) $$\begin{array}{cc} & E\left[G\cdot M_{\hat{\ell },1}^{\prime }\left(W_{\sigma };1\right)\right]=\sigma /\delta \;. \end{array}$$

Next, we show how to use (14) to derive an equivalent system of equations based on $W_{\sigma }$. Starting with (14c), we have

(15) $$\begin{array}{c} E\left[G\cdot M_{\hat{\ell },1}^{\prime }\left(W_{\sigma };1\right)\right]=\frac{1}{\sigma }\;\int \int \;u\;M_{\hat{\ell },1}^{\prime }\left(u+z;1\right)\phi_{\sigma }\left(u\right)p_{SY}\left(z\right)dudz, \end{array}$$

where $\phi_{\sigma }\left(u\right):=p_{\sigma G}\left(u\right)=\frac{1}{\sigma \sqrt{2\pi }}e^{-\frac{u^{2}}{2\sigma^{2}}}$. Since it holds that $\phi_{\sigma }\left(u\right)=\frac{-\sigma^{2}}{u}\phi_{\sigma }^{\prime }\left(u\right)$, using (A74), it follows that

(16) $$\begin{array}{c} \begin{array}{cc} \;E\left[G\cdot M_{\hat{\ell },1}^{\prime }\left(W_{\sigma };1\right)\right] & =-\sigma \;\int \int \;M_{\hat{\ell },1}^{\prime }\left(u+z;1\right)\phi_{\sigma }^{\prime }\left(u\right)p_{SY}\left(z\right)dudz\\ \; & =-\sigma \;\int \int \;M_{\hat{\ell },1}^{\prime }\left(w;1\right)\phi_{\sigma }^{\prime }\left(u\right)p_{SY}\left(w-u\right)dudw\\ & =-\sigma \int M_{\hat{\ell },1}^{\prime }\left(w;1\right)p_{W_{\sigma }}^{\prime }\left(w\right)dw, \end{array} \end{array}$$

where in the last step we use

$$p_{W_{\sigma }}^{\prime }\left(w\right)=\int \phi_{\sigma }^{\prime }\left(u\right)p_{SY}\left(w-u\right)\;du.$$

Therefore, we have by (16) that

(17) $$\begin{array}{c} E\left[G\cdot M_{\hat{\ell },1}^{\prime }\left(W_{\sigma };1\right)\right]=-\sigma \;E\left[M_{\hat{\ell },1}^{\prime }\left(W_{\sigma };1\right)\xi_{W_{\sigma }}\left(W_{\sigma }\right)\right]. \end{array}$$

This combined with (14c) gives $E\left[M_{\hat{\ell },1}^{\prime }\left(W_{\sigma };1\right)\xi_{W_{\sigma }}\left(W_{\sigma }\right)\right]=-1/\delta .$ Second, multiplying (14c) with $\sigma^{2}$ and adding it to (14a) yields that,

(18) $$\begin{array}{cc} E\left[W_{\sigma }\cdot M_{\hat{\ell },1}^{\prime }\left(W_{\sigma };1\right)\right] & =\sigma^{2}/\delta , \end{array}$$

Putting these together, we conclude with the following system of equations which is equivalent to (14),

(19a) $$\begin{array}{cc} & E\left[W_{\sigma }\cdot M_{\hat{\ell },1}^{\prime }\left(W_{\sigma };1\right)\right]=\sigma^{2}/\delta \;, \end{array}$$

(19b) $$\begin{array}{cc} & E\left[\;{\left(M_{\hat{\ell },1}^{\prime }\left(W_{\sigma };1\right)\right)}^{2}\;\right]=\sigma^{2}/\delta \;, \end{array}$$

(19c) $$\begin{array}{cc} & E\left[M_{\hat{\ell },1}^{\prime }\left(W_{\sigma };1\right)\xi_{W_{\sigma }}\left(W_{\sigma }\right)\right]=-1/\delta \;. \end{array}$$

Note that, for σ>0, $\xi_{W_{\sigma }}=p_{W_{\sigma }}^{\prime }/p_{W_{\sigma }}$ exists everywhere. This is because for all w∈R: $p_{W_{\sigma }}\left(w\right)>0$ and $p_{W_{\sigma }}\left(\cdot \right)$ is continuously differentiable. Combining (19a) and (19c), we derive the following equation which holds for $\alpha_{1},\alpha_{2}\in R$,

$$\begin{array}{cc} E\left[\left(\alpha_{1}W_{\sigma }+\alpha_{2}\xi_{W_{\sigma }}\left(W_{\sigma }\right)\right)\cdot M_{\hat{\ell },1}^{\prime }\left(W_{\sigma };1\right)\right] & =\alpha_{1}\sigma^{2}/\delta -\alpha_{2}/\delta . \end{array}$$

By Cauchy–Schwarz inequality, we have that

(20) $$\begin{array}{cc} \; & {\left(E\left[\left(\alpha_{1}W_{\sigma }+\alpha_{2}\xi_{W_{\sigma }}\left(W_{\sigma }\right)\right)\cdot M_{\hat{\ell },1}^{\prime }\left(W_{\sigma };1\right)\right]\right)}^{2}\leq \;\\ & \;\;E\left[{\left(\alpha_{1}W_{\sigma }+\alpha_{2}\xi_{W_{\sigma }}\left(W_{\sigma }\right)\right)}^{2}\right]E\left[{\left(M_{\hat{\ell },1}^{\prime }\left(W_{\sigma };1\right)\right)}^{2}\right]. \end{array}$$

Using the fact that $E[W_{\sigma }\xi_{W_{\sigma }}\left(W_{\sigma }\right)]=-1$ (by integration by parts), $E\left[{\left(\xi_{W_{\sigma }}\left(W_{\sigma }\right)\right)}^{2}\right]=I\left(W_{\sigma }\right)$, $E\left[W_{\sigma }^{2}\right]=\sigma^{2}+1$ and (19b), the right hand side of (20) is equal to

$$\begin{array}{c} \left(\alpha_{1}^{2}\left(\sigma^{2}+1\right)+\alpha_{2}^{2}\;I\left(W_{\sigma }\right)-2\alpha_{1}\alpha_{2}\right)\;\sigma^{2}/\delta . \end{array}$$

Therefore, we conclude with the following inequality for σ,

(21) $$\begin{array}{c} \delta \sigma^{2}\left(\alpha_{1}^{2}\left(\sigma^{2}+1\right)+\alpha_{2}^{2}\;I\left(W_{\sigma }\right)-2\alpha_{1}\alpha_{2}\right)\geq {\left(\alpha_{1}\sigma^{2}-\alpha_{2}\right)}^{2}, \end{array}$$

which holds for all $\alpha_{1},\alpha_{2}\in R$. In particular, (21) holds for the following choice of values for $\alpha_{1}$ and $\alpha_{2}$:

$$\begin{array}{c} \alpha_{1}=\frac{1-\sigma^{2}I\left(W_{\sigma }\right)}{\delta (\sigma^{2}I\left(W_{\sigma }\right)+I\left(W_{\sigma }\right)-1)},\;\;\;\;\;\alpha_{2}=\frac{1}{\delta (\sigma^{2}I\left(W_{\sigma }\right)+I\left(W_{\sigma }\right)-1)}. \end{array}$$

(The choice above is motivated by the result of Section 3.2; see Theorem 3). Rewriting (21) with the chosen values of $\alpha_{1}$ and $\alpha_{2}$ yields the following inequality,

(22) $$\begin{array}{c} \frac{1}{\delta }\leq \frac{\sigma^{2}\left(\sigma^{2}I\left(W_{\sigma }\right)+I\left(W_{\sigma }\right)-1\right)}{1+\sigma^{2}\left(\sigma^{2}I\left(W_{\sigma }\right)-1\right)}=\kappa \left(\sigma \right), \end{array}$$

where on the right-hand side above, we recognize the function κ defined in the theorem.

Next, we use (22) to show that $\sigma_{\mathrm{opt}}$ defined in (12) yields a lower bound on the achievable value of σ. For the sake of contradiction, assume that $\sigma <\sigma_{\mathrm{opt}}$. By the above, 1/δ≤κ(σ). Moreover, by the definition of $\sigma_{\mathrm{opt}}$, we must have that 1/δ<κ(σ). Since κ(0)=0 and κ(·) is a continuous function we conclude that for some $\sigma_{1}\in \left(0,\sigma \right)$, it holds that $\kappa (\sigma_{1})=1/\delta$. Therefore, for $\sigma_{1}<\sigma_{\mathrm{opt}}$, we have $\kappa (\sigma_{1})=1/\delta$, which contradicts the definition of $\sigma_{\mathrm{opt}}$. This proves that $\sigma \geq \sigma_{\mathrm{opt}}$, as desired.

To complete the proof, it remains to show that the equation κ(σ)=1/δ admits a solution for all δ>1. For this purpose, we use the continuous mapping theorem and the fact that the Fisher information is a continuous function [54]. Recall that, for two independent and non-constant random variables, it holds that I(X+Y)<I(X) [53] (Eq. 2.18). Since *G* and SY are independent random variables, we find that I(σG+SY)<I(SY) which implies that I(σG+SY) is uniformly bounded for all values of σ. Therefore,

$$\lim_{\sigma \to 0}\kappa \left(\sigma \right)=\lim_{\sigma \to 0}\frac{\sigma^{2}\left(\sigma^{2}I\left(W_{\sigma }\right)+I\left(W_{\sigma }\right)-1\right)}{1+\sigma^{2}\left(\sigma^{2}I\left(W_{\sigma }\right)-1\right)}=0.$$

Furthermore, $\sigma^{2}I\left(\sigma G+SY\right)=I\left(G+\frac{1}{\sigma }SY\right)\to I\left(G\right)=1$ when σ→∞. Hence,

$$\lim_{\sigma \to \infty }\kappa \left(\sigma \right)=\lim_{\sigma \to \infty }\frac{\sigma^{2}\left(\sigma^{2}I\left(W_{\sigma }\right)+I\left(W_{\sigma }\right)-1\right)}{1+\sigma^{2}\left(\sigma^{2}I\left(W_{\sigma }\right)-1\right)}=1.$$

Note that $\sigma^{2}I\left(\sigma G+SY\right)<\sigma^{2}I\left(\sigma G\right)=1$, which further yields that κ(σ)<1 for all σ≥0. Finally, since I(·) is a continuous function, we deduce that range of $\kappa :R^{+}\cup 0\to R$ is [0,1), implying the *existence* of a solution to (12) for all δ>1. This completes the proof of Theorem 2. □

**A useful closed-form bound on the best achievable performance:** In general, determining $\sigma_{\mathrm{opt}}$ requires computing the Fisher information of the random variable σG+SY for σ>0. If the probability distribution of SY is continuously differentiable (e.g., logistic model; see Appendix C.1), then we obtain the following simplified bound.

**Corollary** **1.** (Closed-form Lower Bound on $\sigma_{\mathrm{opt}}$). *Let $p_{SY}:R\to R$ be the probability distribution of SY. If $p_{SY}\left(x\right)$ is differentiable for all x∈R, then,*

(23) $$\begin{array}{c} \sigma_{\mathrm{opt}}^{2}\geq \frac{1}{\left(\delta -1)(I(SY)-1\right)}\;. \end{array}$$

**Proof.**  Based on Theorem 2, the following equation holds for $\sigma =\sigma_{\mathrm{opt}}$

$$\frac{1}{\delta }=\kappa \left(\sigma \right)$$

or, equivalently, by rewriting the right-hand side,

(24) $$\begin{array}{c} \frac{1}{\delta }=1-\frac{1}{\frac{1}{1-\sigma^{2}I\left(W_{\sigma }\right)}-\sigma^{2}}\;. \end{array}$$

Define the following function

$$\begin{array}{c} h\left(x\right):=1-\frac{1}{\frac{1}{1-\sigma^{2}x}-\sigma^{2}}\;. \end{array}$$

The function *h* is increasing in the region $R_{\sigma }=\left\{z:z>\sigma^{-2}-\sigma^{-4}\right\}.$ According to Stam’s inequality [55], for two independent random variables *X* and *Y* with continuously differentiable $p_{X}$ and $p_{Y}$, it holds that

$$I\left(X+Y\right)\leq \frac{I(X)\cdot I(Y)}{I(X)+I(Y)},$$

where equality is achieved if and only if *X* and *Y* are independent Gaussian random variables. Therefore, since by assumption $p_{SY}$ is differentiable on the real line, Stam’s inequality yields

(25) $$\begin{array}{c} I(W_{\sigma })=I(\sigma \;G+SY)\leq \frac{I(\sigma \;G)\cdot I(SY)}{I(\sigma \;G)+I(SY)}\;. \end{array}$$

Next, we prove that for all σ>0, both sides of (25) are in the region $R_{\sigma }$. First, we prove that $I\left(W_{\sigma }\right)\in R_{\sigma }$. By Cramer–Rao bound (e.g., see [53] (Eq. 2.15)) for Fisher information of a random variable *X*, we have that $I\left(X\right)\geq 1/\left(\mathrm{Var}\left[X\right]\right)$. In addition, for the random variable $W_{\sigma }$, we know that $\mathrm{Var}\left[W_{\sigma }\right]=1+\sigma^{2}-{\left(E\left[SY\right]\right)}^{2}$, thus

(26) $$\begin{array}{c} I\left(W_{\sigma }\right)\geq \frac{1}{1+\sigma^{2}-{\left(E\left[SY\right]\right)}^{2}}. \end{array}$$

Using the relation ${\left(E\left[SY\right]\right)}^{2}\leq E\left[S^{2}\right]E\left[Y^{2}\right]=1$, one can check that the following inequality holds:

(27) $$\begin{array}{c} \frac{1}{1+\sigma^{2}-{\left(E\left[SY\right]\right)}^{2}}\geq \sigma^{-2}-\sigma^{-4}. \end{array}$$

Therefore, from (26) and (27), we derive that $I\left(W_{\sigma }\right)\in R_{\sigma }$ for all σ>0. Furthermore, by the inequality in (25) and the definition of $R_{\sigma }$ it directly follows that for all σ>0

$$\frac{I(\sigma \;G)\;I(SY)}{I(\sigma \;G)+I(SY)}\in R_{\sigma }\;.$$

Finally, noting that h(·) is increasing in $R_{\sigma }$, combined with (25), we have

$$\frac{1}{\delta }=h\left(I(W_{\sigma })\right)\leq h\left(\frac{I(\sigma \;G)\cdot I(SY)}{I(\sigma \;G)+I(SY)}\right),$$

which after using the relation $I\left(\sigma \;G\right)=\sigma^{-2}$ and further simplification yields the inequality in the statement of the corollary. □

**Figure A2:**
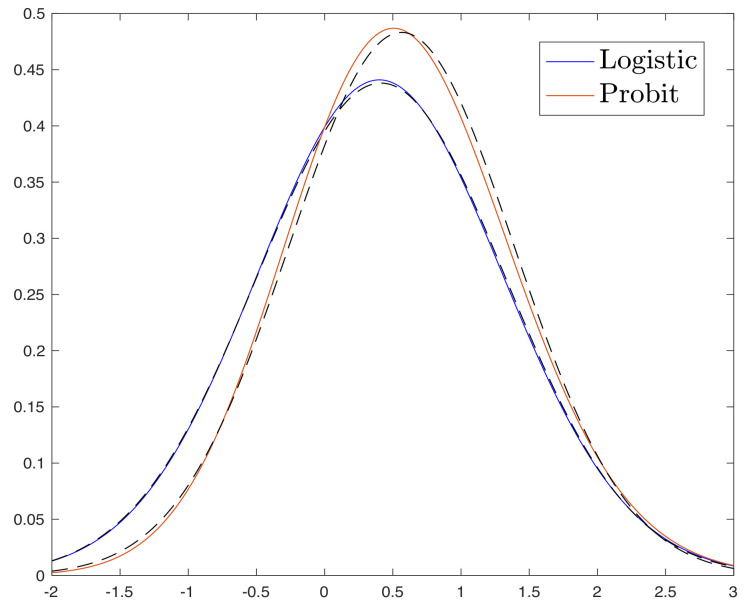
Probability distribution function of SY for the logistic and Probit models ($∥x_{0}{∥}_{2}=1$) compared with the probability distribution function of the Gaussian random variable (dashed lines) with the same mean and variance i.e., N(E[SY],Var[SY]).

**Figure A4:**
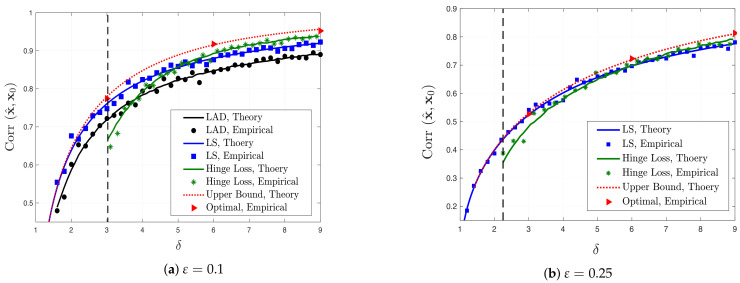
Comparisons between analytical and empirical results for the least-squares (LS), least-absolute deviations and hinge loss functions along with the upper bound on performance and the empirical performance of optimal loss function as in Theorem 3, for noisy-signed measurement model with ε=0.1 (**a**) and ε=0.25 (**b**). The vertical dashed lines are evaluated by (A59) and represent $\delta_{f_{\varepsilon }}^{☆}\approx 3$ and 2.25 for ε=0.1 and 0.25, respectively.

The proof of the corollary reveals that (23) holds with equality when SY is Gaussian. In Appendix C.1, we compute $p_{SY}$ for the logistic and the Probit models with $∥x_{0}{∥}_{2}=1$ and numerically show that it is close to the density of a Gaussian random variable. Consequently, the lower bound of Corollary 1 is almost exact when measurements are obtained according to the logistic and Probit models (see Figure A2 in the Appendix C).

### 3.2. On the Optimal Loss Function

It is natural to ask whether there exists a loss function that attains the bound of Theorem 2. If such a loss function exists, then we say it is *optimal* in the sense that it maximizes the correlation performance among all convex loss functions in (3).

Our next theorem derives a candidate for the optimal loss function, which we denote $\ell_{\mathrm{opt}}$. Before stating the result, we provide some intuition about the proof which builds on Theorem 2. The critical observation in the proof of Theorem 2 is that the effective noise $\sigma_{\hat{\ell }}$ of $\hat{\ell }$ is minimized (i.e., it attains the value $\sigma_{\mathrm{opt}}$) if the Cauchy–Schwartz inequality in (20) holds with equality. Hence, we seek $\hat{\ell }=\ell_{\mathrm{opt}}$ so that for some c∈R,

(28) $$\begin{array}{c} M_{\ell_{\mathrm{opt}},1}^{\prime }\left(w;1\right)=c\left(\alpha_{1}w+\alpha_{2}\cdot \xi_{W_{\mathrm{opt}}}\left(w\right)\right). \end{array}$$

By choosing c=−1, integrating and ignoring constants irrelevant to the minimization of the loss function, the previous condition is equivalent to the following $M_{\ell_{\mathrm{opt}}}\left(w;1\right)=-\alpha_{1}w^{2}/2-\alpha_{2}\log\left(p_{W_{\mathrm{opt}}}\left(w\right)\right).$ It turns out that this condition can be “inverted” to yield the explicit formula for $\ell_{\mathrm{opt}}$ as, $\ell_{\mathrm{opt}}\left(w\right)=-M_{\alpha_{1}q+\alpha_{2}\log\left(p_{W_{\mathrm{opt}}}\right)}\left(w;1\right).$ Of course, one has to properly choose $\alpha_{1}$ and $\alpha_{2}$ to make sure that this function satisfies the system of equations in (19) with $\sigma =\sigma_{\mathrm{opt}}$. The correct choice is specified in the theorem below. The proof is deferred to Appendix D.1.

**Theorem** **3.** (Optimal Loss Function). *Recall the definition of $\sigma_{\mathrm{opt}}$ in (12). Define the random variable $W_{\mathrm{opt}}:=\sigma_{\mathrm{opt}}\;G+SY$ and let $p_{W_{\mathrm{opt}}}$ denote its density. Consider the following loss function $\ell_{\mathrm{opt}}:R\to R$*

(29) $$\begin{array}{c} \ell_{\mathrm{opt}}\left(w\right)=-M_{\alpha_{1}q+\alpha_{2}\log\left(p_{W_{\mathrm{opt}}}\right)}\left(w;1\right), \end{array}$$

*where $q\left(x\right)=x^{2}/2$ and*

(30) $$\begin{array}{c} \begin{array}{cc} \alpha_{1} & =\frac{1-\sigma_{\mathrm{opt}}^{2}I\left(W_{\mathrm{opt}}\right)}{\delta (\sigma_{\mathrm{opt}}^{2}I\left(W_{\mathrm{opt}}\right)+I\left(W_{\mathrm{opt}}\right)-1)},\\ \alpha_{2} & =\frac{1}{\delta (\sigma_{\mathrm{opt}}^{2}I\left(W_{\mathrm{opt}}\right)+I\left(W_{\mathrm{opt}}\right)-1)}. \end{array} \end{array}$$

*If $\ell_{\mathrm{opt}}$ defined as in (29) is convex and the equation κ(σ)=1/δ has a unique solution, then $\sigma_{\ell_{\mathrm{opt}}}=\sigma_{\mathrm{opt}}$.*

In general, there is no guarantee that the function $\ell_{\mathrm{opt}}\left(\cdot \right)$ as defined in (29) is convex. However, if this is the case, the theorem above guarantees that it is optimal (Strictly speaking, the performance is optimal among all convex loss functions *ℓ* for which (8) has a unique solution as required by Theorem 2.). A *sufficient* condition for $\ell_{\mathrm{opt}}\left(w\right)$ to be convex is provided in Appendix D.2. Importantly, in Appendix D.2.1, we show that this condition holds for observations following the signed model. Thus, for this case, the resulting function is convex. Although we do *not* prove the convexity of optimal loss function for the logistic and Probit models, our numerical results (e.g., see Figure 3b) suggest that this is the case. Concretely, we conjecture that the loss function $\ell_{\mathrm{opt}}$ is convex for logistic and Probit models, and therefore by Theorem 3 its performance is optimal.

## 4. Special Cases

### 4.1. Least-Squares

By choosing $\ell \left(t\right)={\left(t-1\right)}^{2}$ in (3), we obtain the standard least-squares estimate. To see this, note that since $y_{i}=\pm 1$, it holds for all *i* that ${\left(y_{i}a_{i}^{T}x-1\right)}^{2}={\left(y_{i}-a_{i}^{T}x\right)}^{2}.$ Thus, $\hat{x}$ is minimizing the sum of squares of the residuals:

(31) $$\begin{array}{c} \hat{x}=\arg\min_{x}\sum {\left(y_{i}-a_{i}^{T}x\right)}^{2}. \end{array}$$

For this choice of a loss function, we can solve the equations in (8) in closed form. Furthermore, the equations have a (unique, bounded) solution for any δ>1 provided that E[SY]>0. The final result is summarized in the corollary below (see Appendix F.1 for the proof).

**Corollary** **2.** (Least-squares). *Assume data generated from the binary model and δ>1. For the label function assume that E[SY]>0 in the notation of (7). Let $\hat{x}$ be as in (41). Then, in the limit of m,n→+∞, m/n→δ, Equations (9) and (10) hold with probability one with α and μ given as follows:*

(32) $$\begin{array}{cc} \mu & =E[SY], \end{array}$$

(33) $$\begin{array}{cc} \alpha & =\sqrt{1-{\left(E[SY]\right)}^{2}}\cdot \sqrt{\frac{1}{\delta -1}}\;. \end{array}$$

Corollary 2 appears in [43] (see also [40,41,56] and Appendix F for an interpretation of the result). However, these previous works obtain results that are limited to least-squares loss. In contrast, our results are general and LS prediction is obtained as a simple corollary of our general Theorem 1. Moreover, our study of fundamental limits allows us to quantify the sub-optimality gap of least-square (LS) as follows.

**On the Optimality of LS.** On the one hand, Corollary 2 derives an explicit formula for the effective noise variance $\sigma_{\mathrm{LS}}=\alpha /\mu$ of LS in terms of E[YS] and δ. On the other hand, Corollary 1 provides an explicit lower bound on the optimal value $\sigma_{\mathrm{opt}}$ in terms of I(SY) and δ. Combining the two, we conclude that

$$\frac{\sigma_{\mathrm{LS}}^{2}}{\sigma_{\mathrm{opt}}^{2}}\leq \xi :=\left(I\left(SY\right)-1\right)\frac{1-{\left(E\left[SY\right]\right)}^{2}}{{\left(E\left[SY\right]\right)}^{2}}\;.$$

In terms of correlation,

$$\begin{array}{c} \frac{{\mathrm{corr}}_{\mathrm{opt}}}{{\mathrm{corr}}_{\mathrm{LS}}}=\sqrt{\frac{1+\sigma_{\mathrm{LS}}^{2}}{1+\sigma_{\mathrm{opt}}^{2}}}\leq \frac{\sigma_{\mathrm{LS}}}{\sigma_{\mathrm{opt}}}\leq \sqrt{\xi }\;, \end{array}$$

where the first inequality follows from the fact that $\sigma_{\mathrm{LS}}\geq \sigma_{\mathrm{opt}}.$ Therefore, the performance of LS is at least as good as $\frac{1}{\sqrt{\xi }}$ times the optimal one. In particular, assuming $∥x_{0}∥=1$ and for logistic and Probit models (for which Corollary 1 holds), we can explicitly compute $\frac{1}{\sqrt{\xi }}=0.9972\;\mathrm{and}\;0.9804$, respectively. However, we recall that for large $∥x_{0}∥$ logistic and Probit models approach the signed model, and, as Figure 1a demonstrates, LS becomes suboptimal.

Another interesting consequence of combining Corollaries 1 and 2 is that LS would be optimal if SY were a Gaussian random variable. To see this, recall from Corollary 1 that, if SY is Gaussian, then:

$$\sigma_{\mathrm{opt}}^{2}=\frac{1}{\left(\delta -1)(I(SY)-1\right)}.$$

However, for SY Gaussian, we can explicitly compute I(SY)=1/Var[SY], which leads to

$$\sigma_{\mathrm{opt}}^{2}=\frac{1-{\left(E\left[SY\right]\right)}^{2}}{{\left(E\left[SY\right]\right)}^{2}\left(\delta -1\right)}.$$

The right hand side is exactly $\sigma_{\mathrm{LS}}^{2}$. Therefore, the optimal performance is achieved by the square loss function if SY is a Gaussian random variable. Remarkably, for logistic and Probit models with small SNR (i.e., small $∥x_{0}∥$), density of SY is close to the density of a normal random variable (see Figure A2 in the Appendix C), implying the optimality of LS for these models.

### 4.2. Logistic and Hinge Loss

Theorem 1 only holds in regimes for which the set of minimizers of (3) is bounded. As we show here, this is not always the case. Specifically, consider non-negative loss functions ℓ(t)≥0 with the property $\lim_{t\to +\infty }\ell \left(t\right)=0$. For example, the hinge, exponential and logistic loss functions all satisfy this property. Now, we show that for such loss functions the set of minimizers is unbounded if $\delta <\delta_{f}^{☆}$ for some appropriate $\delta_{f}^{☆}>2$. First, note that the set of minimizers is unbounded if the following condition holds:

(34) $$\begin{array}{c} \exists x_{s}\neq 0\;\mathrm{such}\;\mathrm{that}\;y_{i}a_{i}^{T}x_{s}\geq 0,\;\forall i\in \left[m\right]. \end{array}$$

Indeed, if (34) holds then $x=c\cdot x_{s}$ with c→+∞, attains zero cost in (3); thus, it is optimal and the set of minimizers is unbounded. To proceed, we rely on a recent result by Candes and Sur [44] who proved that (34) holds iff (To be precise, Candes and Sur [44] proved the statement for measurements $y_{i},i\in \left[m\right]$ that follow a logistic model. Close inspection of their proof shows that this requirement can be relaxed by appropriately defining the random variable *Y* in (7) (see also [48,49]).)

(35) $$\begin{array}{c} \delta \leq \delta_{f}^{☆}:={\left(\min_{c\in R}E\left[{\left(G+c\;S\;Y\right)}_{-}^{2}\right]\right)}^{-1}, \end{array}$$

where G,S and *Y* are random variables as in (7) and ${\left(t\right)}_{-}:=\min\left\{0,t\right\}$. We highlight that logistic and hinge losses give unbounded solutions in the noisy-signed model with ε=0, since the condition (34) holds for $x_{s}=x_{0}$. However, their performances are comparable to the optimal performance in both logistic and Probit models (see Figure 3a and Figure 4a).

## 5. Extensions to Gaussian-Mixture Models

In this section, we show that our results on sharp asymptotics and lower bounds on error can be extended to include the Gaussian-Mixture model (GMM) presented in Section 1.2. The discussions on the phase transition for the existence of a bounded solution in Section 4.2 applies here as well. We rely on a phase-transition result [49] (Prop. 3.1), which proves that (34) holds if and only if

(36) $$\begin{array}{c} \delta \leq \delta^{☆}:={\left(\min_{t\in R}E\left[{\left(W_{1}+t\;W_{2}\right)}_{-}^{2}\right]\right)}^{-1}, \end{array}$$

where $W_{1}$ and $W_{2}$ are random variables defined in (7) and ${\left(x\right)}_{-}^{2}:={\left(\min\{x,0\}\right)}^{2}$. Therefore, for loss functions satisfying this property, e.g., hinge loss and logistic loss, the solution to (3) is unbounded if and only if $\delta \leq \delta^{☆}.$

### 5.1. System of Equations for GMM

It turns out that, similar to the generative models, the asymptotic performance of (3) for GMM depends on the loss function *ℓ* via its Moreau envelope. Specifically, let $W_{1}$ and $W_{2}$ be independent Gaussian random variables such that

(37) $$\begin{array}{c} W_{1}∼N\left(0,1\right),\;W_{2}∼N\left(r,1\right), \end{array}$$

where $r:=∥x_{0}{∥}_{2}\;>0.$ Consider the following system of non-linear equations in three unknowns (μ,α≥0,λ≥0):

(38a) $$\begin{array}{cc} 0 & =E\left[W_{2}\cdot M_{\ell ,1}^{\prime }\left(\alpha W_{1}+\mu W_{2};\lambda \right)\right], \end{array}$$

(38b) $$\begin{array}{cc} \alpha^{2} & =\;\lambda^{2}\;\delta \;E\left[{\left(M_{\ell ,1}^{\prime }\left(\alpha W_{1}+\mu W_{2};\lambda \right)\right)}^{2}\;\right], \end{array}$$

(38c) $$\begin{array}{cc} \alpha & =\lambda \;\delta \;E\left[W_{1}\cdot M_{\ell ,1}^{\prime }\left(\alpha W_{1}+\mu W_{2};\lambda \right)\right]. \end{array}$$

The expectations above are with respect to the randomness of the random variables $W_{1}$ and $W_{2}$.

As we show shortly, the solution to these equations is tightly connected to the asymptotic behavior of the optimization in (3).

### 5.2. Theoretical Prediction of Error for Convex Loss Functions

**Theorem** **4.** (Asymptotic Prediction). *Assume data generated from the Gaussian-mixture model and assume δ>1 such that the set of minimizers in (3) is bounded and the system of Equation (38) has a unique solution (μ,α,λ), such that μ≠0. Let ${\hat{x}}_{\ell }$ be as in (3) and $\sigma_{\ell }=\alpha /\mu$. Then, in the limit of m,n→+∞, m/n→δ, it holds with probability one that*

(39) $$\begin{array}{c} \lim_{n\to \infty }\mathrm{corr}\left(\;{\hat{x}}_{\ell }\;;\;x_{0}\;\right)=\frac{\mu }{\sqrt{\mu^{2}+\alpha^{2}}},\;\;\lim_{n\to \infty }E_{\ell }=Q\left(\frac{r}{\sqrt{1+\sigma_{\ell }^{2}}}\right), \end{array}$$

*where $E_{\ell }$ denotes the classification test error defined in (5).*

**Remark** **6** (Proof of Theorem 4). *The high-level steps of the proof of Theorem 4 follow closely the proof of Theorem 1. Particularly, for GMM one can show the correlation of the ERM estimate with the true vector $x_{0}$ is predicted by a system of Equations as in (38), only with $W_{2}$ replaced by a non-gaussian random variable (denoted as SY in Theorem 1). Specifically, by rotational invariance of the Gaussian feature vectors $a_{i}$, we can assume, without loss of generality, that $x_{0}={\left[r,0,0,\ldots ,0\right]}^{T}$. Then, we can can guarantee that with probability one it holds that*

(40) $$\begin{array}{c} \lim_{n\to \infty }{\hat{x}}_{\ell }\left(1\right)=\mu ,\;\;\;\lim_{n\to \infty }\sum_{j=2}^{n}{\hat{x}}_{\ell }^{2}\left(j\right)=\alpha^{2}, \end{array}$$

*where μ and α are specified by (38). To see how this implies (39), we argue as follows. Recalling that $x|y∼N(yx_{0},I)$, we have*

$$y\langle {\hat{x}}_{\ell },a\rangle ∼N\left(r{\hat{x}}_{\ell }\left(1\right),{∥{\hat{x}}_{\ell }∥}_{2}^{2}\;\right).$$

*Using this and (40) leads to the asymptotic value of correlation and classification error as presented in (39).*

**Remark** **7.** (On the Uniqueness of Solutions to Equation (38)) Our results in proving the uniqueness of solutions to the equations for generative models (8) in Proposition 1, extend to GMM. Noting that $W_{2}∼N\left(r,1\right)$ in (38) plays the role of SY in (8), we straightforwardly deduce the following result for uniqueness of solutions to (38).

**Proposition** **2.** Assume that the loss function ℓ:R→R has the following properties: (i) it is proper strictly convex; and (ii) it is continuously differentiable and its derivative $\ell^{\prime }$ is such that $\ell^{\prime }\left(0\right)\neq 0$. The following statement is true. For any δ>1, if the system of equations in (38) has a bounded solution, then it is unique.

### 5.3. Special Case: Least-Squares

By choosing $\ell \left(t\right)={\left(t-1\right)}^{2}$ in (3), we obtain the standard least-squares estimate. To see this, note that since $y_{i}=\pm 1$, it holds for all *i* that ${\left(y_{i}a_{i}^{T}x-1\right)}^{2}={\left(y_{i}-a_{i}^{T}x\right)}^{2}.$

Thus, the estimator ${\hat{x}}_{LS}$ is minimizing the sum of squares of the residuals:

(41) $$\begin{array}{c} {\hat{x}}_{LS}=\arg\min_{x}\sum {\left(y_{i}-a_{i}^{T}x\right)}^{2}. \end{array}$$

For the choice $\ell \left(t\right)={\left(t-1\right)}^{2}$, it turns out that we can solve the equations in (38) in closed form. The final result is summarized in the corollary below and proved in Appendix G.1.

**Corollary** **3.** (Least-Squares). *Let ${\hat{x}}_{LS}$ be as in (31) and δ>1. Then, in the limit of m,n→+∞, m/n→δ, Equation (39) holds with probability one with $\sigma_{LS}^{2}$ given as follows:*

(42) $$\begin{array}{c} \sigma_{LS}^{2}=\frac{1+r^{2}}{r^{2}}\cdot \frac{1}{\left(\delta -1\right)}. \end{array}$$

### 5.4. Optimal Risk for GMM

Next, we characterize the best achievable classification error by different choices of loss function. Considering (39), we see that an optimal choice of *ℓ* is the one that minimizes $\sigma_{\ell }^{2}$. The next theorem characterizes the best achievable $\sigma_{\ell }$ among convex loss functions by deriving an equivalent set of equations to (38) and combining them with proper coefficients. Similar to the proof of Theorem 2, a key step in the proof is properly setting up a Cauchy–Schwarz inequality that exploits the structure of the new set of equations. The proof is deferred to Appendix G.2.

**Theorem** **5.** (Lower Bound on Risk). *Under the assumptions of Theorem 4, the following inequality holds for the effective risk parameter ($\sigma_{\ell }$) of a loss function ℓ:*

(43) $$\begin{array}{c} \lim_{n\to \infty }\sigma_{\ell }^{2}\geq \sigma_{☆}^{2}:=\frac{1+r^{2}}{r^{2}}\cdot \frac{1}{\delta -1} \end{array}$$

**Remark** **8.** (Optimality of Least-squares for GMM). *Theorem 5 provides a lower bound for the asymptotic value of $\sigma_{\ell }$ which holds for all δ>1 and r>0. This result together with Corollary 3 implies that least-squares achieves the least value of risk (i.e., $\sigma_{\ell }$ and $E_{\ell }$) for all δ>1 and r>0 among all convex loss functions ℓ for which the set of minimizers in (3) is bounded.*

## 6. Numerical Experiments

In this section, we present numerical simulations that validate the predictions of Theorems 1–5. To begin, we use the following three popular models as our case study: signed, logistic and Probit. We generate random measurements according to (1). Without loss of generality (due to rotational invariance of the Gaussian measure), we set $x_{0}={\left[1,0,\ldots ,0\right]}^{T}$. We then obtain estimates ${\hat{x}}_{\ell }$ of $x_{0}$ by numerically solving (3) and measure performance by the correlation value $\mathrm{corr}\left(\;{\hat{x}}_{\ell }\;;\;x_{0}\;\right)$. Throughout the experiments, we set n=128 and the recorded values of correlation are averages over 25 independent realizations. For each label function, we first provide plots that compare results of Monte Carlo simulations to the asymptotic predictions for loss functions discussed in Section 4, as well as to the optimal performance of Theorem 2. We next present numerical results on optimal loss functions. To empirically derive the correlation of optimal loss function, we run gradient descent-based optimization with 1000 iterations. As a general comment, we note that, despite being asymptotic, our predictions appear accurate even for relatively small problem dimensions. For the analytical predictions, we apply Theorem 1. In particular, for solving the system of non-linear equations in (3), we empirically observe (see also [15,47] for similar observation) that, if a solution exists, then it can be efficiently found by the following fixed-point iteration method. Let $v:={\left[\mu ,\alpha ,\lambda \right]}^{T}$ and $F:R^{3}\to R^{3}$ be such that (3) is equivalent to v=F(v). With this notation, we initialize $v=v_{0}$ and for k≥1 repeat the iterations $v_{k+1}=F\left(v_{k}\right)$ until convergence.

**Logistic model.** For the logistic model, comparison between the predicted values and the numerical results is illustrated in Figure 3a. Results are shown for LS, logistic and hinge loss functions. Note that minimizing the logistic loss corresponds to the maximum-likelihood estimator (MLE) for logistic model. An interesting observation in Figure 3a is that in the high-dimensional setting (finite δ) LS has comparable (if not slightly better) performance to MLE. Additionally, we observe that in this model, performance of LS is almost the same as the best possible performance derived according to Theorem 2. This confirms the analytical conclusion of Section 4.1. The comparison between the optimal loss function as in Theorem 3 and other loss functions is illustrated in Figure 3b. We note the obvious similarity between the shapes of optimal loss functions and LS which further explains the similarity between their performance.

**Probit model.** Theoretical predictions for the performance of hinge and LS loss functions are compared with the empirical results and optimal performance of Theorem 2 in Figure 4a. Similar to the logistic model, in this model, LS also outperforms hinge loss and its performance resembles the performance of optimal loss function derived according to Theorem 3. Figure 4b illustrates the shapes of LS, hinge loss and the optimal loss functions for the Probit model. The obvious similarity between the shape of LS and optimal loss functions for all values of δ explains the close similarity of their performance. 

Additionally, by comparing the LS performance for the three models in Figure 1a, Figure 3a and Figure 4a, it is clear that higher (respectively, lower) correlation values are achieved for signed (respectively, logistic) measurements. This behavior is indeed predicted by Corollary 2: correlation performance is higher for higher values of μ=E[SY]. It can be shown that, for the signed, probit and logistic models (with $∥x_{0}{∥}_{2}=1$), we have $\mu =\sqrt{2/\pi },\sqrt{1/\pi }\;\mathrm{and}\;0.4132$, respectively.

**Optimal loss function.** By putting together Theorems 2 and 3, we obtain a method on deriving the optimal loss function for generative binary models. This requires the following steps.

1. Find $\sigma_{\mathrm{opt}}$ by solving (12).
2. Compute the density of $W_{\mathrm{opt}}=\sigma_{\mathrm{opt}}G+SY$.
3. Compute $\ell_{\mathrm{opt}}$ according to (29).

Note that computing $\sigma_{\mathrm{opt}}$ needs the density function $p_{W}$ of the random variable W=σG+SY. In principle $p_{W}$ can be calculated as the convolution of the Gaussian density with the pdf $p_{SY}$ of SY. Moreover, it follows from the recipe above that the optimal loss function depends on δ in general. This is because $\sigma_{\mathrm{opt}}$ itself depends on δ via (12).

### Numerical Experiments for GMM

Theorem 5 implies the optimality of least-squares among convex loss functions in the under-parameterized regime δ>1. In Figure 2, we demonstrate the classification risk of least-squares alongside other well-known loss functions LAD and logistic, for r=1. Solid lines correspond to the theoretical predictions of Theorem 4. For least-squares we rely on the result of Corollary 3 and for LAD and logistic loss, the system of equations are solved by iterating over the equations, where we observe that after relatively small number of iterations the triple (μ,α,λ) converges to $\left(\mu^{☆},\alpha^{☆},\lambda^{☆}\right)$. We use $10^{5}$ and $10^{3}$ samples to compute the expectations in (38) for LAD and logistic loss, respectively. After deriving $\sigma_{\ell }=\alpha /\mu$, the classification risk $E_{\ell }$ is obtained according to the formula in (39). Dots correspond to the empirical evaluations of the classification risk of loss functions for n=60 and for different values of δ=m/n>1. The resulting numbers are averaged over 30 independent experiments. As is observed, the empirical results closely follow the theoretical predictions of Theorem 4. Furthermore, as predicted by Theorem 5, least-squares has the minimum expected classification risk among other convex loss functions and for all δ>1.

## 7. Conclusions

We derive theoretical predictions for the generalization error of estimators obtained by ERM for generative binary models and a Gaussian Mixture model. Furthermore, we use this theoretical characterizations to find the optimal performance and optimal loss function among all convex losses. Although our analysis is true for Gaussian matrices, we empirically show they hold for sub-Gaussian matrices as well. As an exciting future direction, we plan to extend our analysis on sharp asymptotics and optimal loss function to non-isotropic (Gaussian) features with arbitrary covariance. A more challenging, albeit interesting, direction is going beyond (binary) linear models studied in this paper, by considering asymptotics and optimal error for kernel models and neural networks (see [48,57] for partial progress in this direction).

## Figures and Tables

**Table 1:** Theoretical predictions and empirical performance of the optimal loss function for signed model. Empirical results are averaged over 20 experiments for n=128.

δ	2	3	4	5	6	7	8	9
Predicted Performance	0.8168	0.9101	0.9457	0.9645	0.9748	0.9813	0.9855	0.9885
Empirical (Gaussian)	0.8213	0.9045	0.9504	0.9669	0.9734	0.9801	0.9834	0.9873
Empirical (Rademacher)	0.8096	0.9158	0.9490	0.9633	0.9644	0.9768	0.9808	0.9829

**Figure A1:**
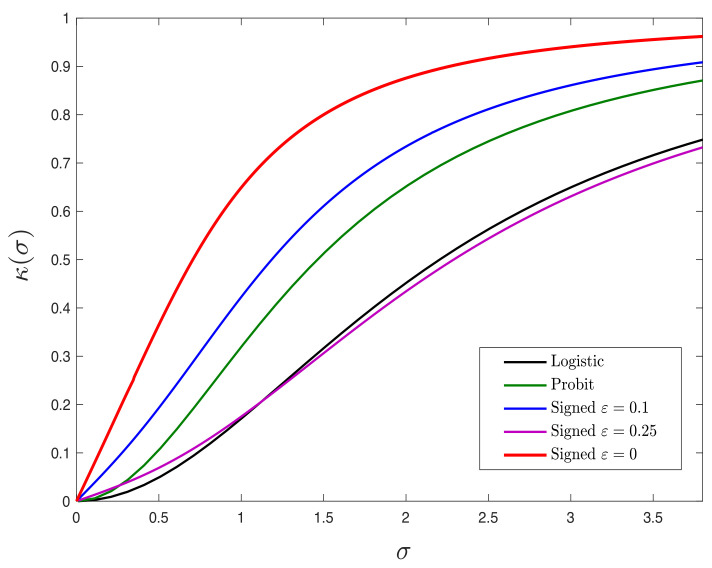
The value of κ(σ) as in Theorem 2 for various measurement models. Since κ(σ) is a monotonic function of σ, the solution to κ(σ)=1/δ determines the minimum possible value of σ.

**Figure A3:**
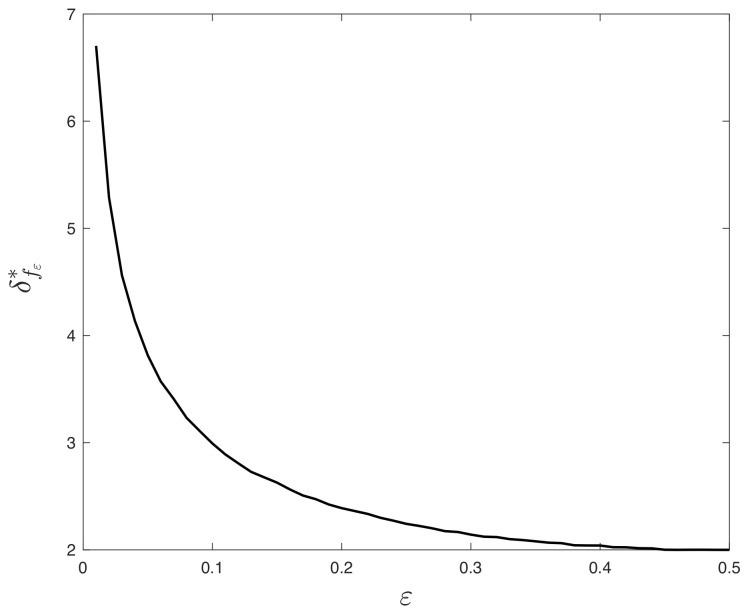
The value of the threshold $\delta_{f_{\varepsilon }}^{☆}$ in (A69) as a function of probability of error ε∈[0,1/2]. For logistic and hinge losses, the set of minimizers in (3) is bounded (as required by Theorem 1) iff $\delta >\delta_{f_{\varepsilon }}^{☆}$.

## Appendix A. Properties of Moreau Envelopes

### Appendix A.1. Derivatives

Recall the definition of the Moreau envelope $M_{\ell }\left(x;\lambda \right)$ and proximal operator ${\mathrm{prox}}_{\ell }\left(x;\lambda \right)$ of a function *ℓ*:

(A1) $$\begin{array}{c} M_{\ell }\left(x;\lambda \right)=\min_{y}\frac{1}{2\lambda }{\left(x-y\right)}^{2}+\ell \left(y\right), \end{array}$$

and ${\mathrm{prox}}_{\ell }\left(x;\lambda \right)=\arg\min_{y}\frac{1}{2\lambda }{\left(x-y\right)}^{2}+\ell \left(y\right)$.

**Proposition** **A1** (Basic properties of $M_{\ell }$ and ${\mathrm{prox}}_{\ell }$ [52]). *Let ℓ:R→R be lower semi-continuous (lsc), proper and convex. The following statements hold for any λ>0.*

(a) The proximal operator ${\mathrm{prox}}_{\ell }\left(x;\lambda \right)$ is unique and continuous. In fact, ${\mathrm{prox}}_{\ell }\left(x;\lambda \right)\to {\mathrm{prox}}_{\ell }\left(x^{\prime };\lambda^{\prime }\right)$ whenever $\left(x,\lambda \right)\to \left(x^{\prime },\lambda^{\prime }\right)$ with $\lambda^{\prime }>0$.
(b) The value $M_{\ell }\left(x;\lambda \right)$ is finite and depends continuously on (λ,x), with $M_{\ell }\left(x;\lambda \right)\to f\left(x\right)$ for all x as $\lambda \to 0_{+}$.
(c) *The Moreau envelope function is differentiable with respect to both arguments. Specifically, for all x∈R, the following properties are true:*
  (A2) $$\begin{array}{cc} M_{\ell ,1}^{\prime }\left(x;\lambda \right) & =\frac{1}{\lambda }\left(x-{\mathrm{prox}}_{\ell }\left(x;\lambda \right)\right), \end{array}$$
  (A3) $$\begin{array}{cc} M_{\ell ,2}^{\prime }\left(x;\lambda \right) & =-\frac{1}{2\lambda^{2}}{\left(x-{\mathrm{prox}}_{\ell }\left(x;\lambda \right)\right)}^{2}. \end{array}$$
  *If in addition ℓ is differentiable and $\ell^{{}^{\prime }}$ denotes its derivative, then*
  (A4) $$\begin{array}{cc} M_{\ell ,1}^{\prime }\left(x;\lambda \right) & =\ell^{\prime }\left({\mathrm{prox}}_{\ell }\left(x;\lambda \right)\right), \end{array}$$
  (A5) $$\begin{array}{cc} M_{\ell ,2}^{\prime }\left(x;\lambda \right) & =-\frac{1}{2}(\ell^{\prime }{\left({\mathrm{prox}}_{\ell }\left(x;\lambda \right)\right)}^{2}. \end{array}$$

### Appendix A.2. Alternative Representations of (8)

Replacing the above relations for derivative of $M_{\ell }$ in (8), we can write the equations in terms of the proximal operator. If *ℓ* is differentiable, then Equations (8) can be equivalently written as follows:

(A6a) $$\begin{array}{cc} E\left[Y\;S\cdot \ell^{\prime }\left({\mathrm{prox}}_{\ell }\left(\alpha G+\mu SY;\lambda \right)\right)\right] & =0, \end{array}$$

(A6b) $$\begin{array}{cc} \lambda^{2}\;\delta \;E\left[{\left(\ell^{\prime }\left({\mathrm{prox}}_{\ell }\left(\alpha G+\mu SY;\lambda \right)\right)\right)}^{2}\right] & =\alpha^{2}, \end{array}$$

(A6c) $$\begin{array}{cc} \lambda \;\delta \;E\left[G\cdot \ell^{\prime }\left({\mathrm{prox}}_{\ell }\left(\alpha G+\mu SY;\lambda \right)\right)\right] & =\alpha . \end{array}$$

Finally, if *ℓ* is two times differentiable, then applying integration by parts in Equation (14c) results in the following reformulation of (8c):

(A7) $$\begin{array}{cc} 1 & =\lambda \;\delta \;E\left[\frac{\ell^{\prime \prime }\left({\mathrm{prox}}_{\ell }\left(\alpha G+\mu SY;\lambda \right)\right)}{1+\lambda \;\ell^{\prime \prime }\left({\mathrm{prox}}_{\ell }\left(\alpha G+\mu SY;\lambda \right)\right)}\right]. \end{array}$$

### Appendix A.3. Examples of Proximal Operators

LAD.

For ℓ(t)=|t−1|, the proximal operator admits a simple expression, as follows:

(A8) $$\begin{array}{c} {\mathrm{prox}}_{\ell }\left(x;\lambda \right)=1+H\left(x-1;\lambda \right), \end{array}$$

where

$$H\left(x;\lambda \right)=\left\{\begin{array}{cc} x-\lambda , & \mathrm{if}\;x>\lambda ,\\ x+\lambda , & \mathrm{if}\;x<-\lambda ,\\ 0, & \mathrm{otherwise}. \end{array}\right.$$

is the standard soft-thresholding function.

Hinge Loss.

When ℓ(t)=max{0,1−t}, the proximal operator can be expressed in terms of the soft-thresholding function as follows:

$$\begin{array}{c} {\mathrm{prox}}_{\ell }\left(x;\lambda \right)=1+H\left(x+\frac{\lambda }{2}-1;\frac{\lambda }{2}\right). \end{array}$$

### Appendix A.4. Fenchel–Legendre Conjugate Representation

For a function h:R→R, its Fenchel–Legendre conjugate, $h^{☆}:R\to R$ is defined as:

$$\begin{array}{c} h^{☆}\left(x\right)=\max_{y}\left[xy-h(y)\right]. \end{array}$$

The following proposition relates Moreau Envelope of a function to its Fenchel–Legendre conjugate.

**Proposition** **A2.** *For λ>0 and a function h, we have:*

(A9) $$\begin{array}{c} M_{h}\left(x;\lambda \right)=\frac{q(x)}{\lambda }-\frac{1}{\lambda }{\left(q+\lambda h\right)}^{☆}\left(x\right), \end{array}$$

*where $q\left(x\right)=x^{2}/2.$*

**Proof.** 

$$\begin{array}{cc} M_{h}\left(x;\lambda \right) & =\frac{1}{2\lambda }\min_{y}\left[{\left(x-y\right)}^{2}+2\lambda h\left(y\right)\right]\\ \; & =\frac{x^{2}}{2\lambda }+\frac{1}{2\lambda }\min_{y}\left[y^{2}-2xy+2\lambda h\left(y\right)\right]\\ \; & =\frac{x^{2}}{2\lambda }-\frac{1}{\lambda }\max_{y}\left[xy-\left(y^{2}/2+\lambda h\left(y\right)\right)\right]\\ \; & =\frac{q(x)}{\lambda }-\frac{1}{\lambda }{\left(q+\lambda h\right)}^{☆}\left(x\right). \end{array}$$

□

### Appendix A.5. Convexity of the Moreau Envelope

**Lemma** **A1.** *The function $H:R^{3}\to R$ defined as follows*

(A10) $$\begin{array}{c} H\left(x,v,\lambda \right)=\frac{1}{2\lambda }{\left(x-v\right)}^{2}, \end{array}$$

*is jointly convex in its arguments.*

**Proof.** Note that the function $h\left(x,v\right)={\left(x-v\right)}^{2}$ is jointly convex in (x,v). Thus, its perspective function

$$\lambda \;h\left(x/\lambda ,v/\lambda \right)={\left(x-v\right)}^{2}/\lambda =2H\left(x,v,\lambda \right)$$

is jointly convex in (x,v,λ) [58] (Sec. 2.3.3), which completes the proof. □

**Proposition** **A3.** *(a) Ref. [52] (Prop. 2.22) Let f(x,y) be jointly convex in its arguments. Then, the function $g\left(x\right)=\min_{y}f\left(x,y\right)$ is convex.*
*(b) Ref. [58] (Sec. 3.2.3) Suppose $f_{i}:R\to R$ is a set of concave functions, with i∈A an index set. Then, the function f:R→R defined as $f\left(x\right):=\inf_{i\in A}f_{i}\left(x\right)$ is concave.*

**Lemma** **A2.** Let ℓ:R→R be a lsc, proper, convex function. Then, $M_{\ell }\left(x;\lambda \right)$ is jointly convex in (x,λ).

**Proof.** Recall that

(A11) $$\begin{array}{c} M_{\ell }\left(x;\lambda \right)=\min_{v}\;G\left(a\right):=\frac{1}{2\lambda }{\left(x-v\right)}^{2}+\ell \left(v\right), \end{array}$$

where, for compactness, we let $a\in R^{3}$ denote the triplet (x,v,λ). Now, let $a_{i}=\left(x_{i},v_{i},\lambda_{i}\right),i=1,2$, θ∈(0,1) and $\bar{\theta }:=1-\theta$. With this notation, we may write

$$\begin{array}{cc} G(\theta a_{1}+\bar{\theta }a_{2}) & =H\left(\theta x_{1}+\bar{\theta }x_{2},\theta \lambda_{1}+\bar{\theta }\lambda_{2},\theta v_{1}+\bar{\theta }v_{2}\right)+\ell \left(\theta v_{1}+\bar{\theta }v_{2}\right)\\ & \leq \theta H\left(x_{1},v_{1},\lambda_{1}\right)+\bar{\theta }H\left(x_{2},v_{2},\lambda_{2}\right)+\theta \ell \left(v_{1}\right)+\bar{\theta }\ell \left(v_{2}\right)\\ & =\theta G\left(a_{1}\right)+\bar{\theta }G\left(a_{2}\right). \end{array}$$

For the first equality above, we recall the definition of $H:R^{3}\to R$ in (A10) and the inequality right after follows from Lemma A1 and convexity of *ℓ*. Thus, the function *G* is jointly convex in its arguments. Using this fact, as well as (A11), and applying Proposition A3(a) completes the proof. □

### Appendix A.6. The Expected Moreau-Envelope (EME) Function and its Properties

The performance of the ERM estimator (3) is governed by the system of equations (8) in which the Moreau envelope function $M_{\ell }\left(x;\lambda \right)$ of the loss function *ℓ* plays a central role. More precisely, as already hinted by (8) and becomes clear in Appendix B, what governs the behavior is the function

(A12) $$\begin{array}{c} \left(\alpha >0,\mu ,\tau >0,\gamma >0\right)\mapsto E\left[M_{\ell }\left(\alpha G+\mu SY;\tau /\gamma \right)\right], \end{array}$$

which we call the expected Moreau envelope (EME). Recall here that Y=f(S). Hence, the EME is the key summary parameter that captures the role of both the loss function ℓ:R→R and of the link function f:R→{±1} on the statistical performance of (3).

In this section, we study several favorable properties of the EME. In (A12), the expectation is over $G,S\overset{\mathrm{iid}}{∼}N\left(0,1\right)$. We first study the EME under more general distribution assumptions in Appendix A.6.1, Appendix A.6.2 and Appendix A.6.3 and we then specialize our results to Gaussian random variables *G* and *S* in Appendix A.6.4.

#### Appendix A.6.1. Derivatives

**Proposition** **A4.** *Let ℓ:R→R be a lsc, proper and convex function. Further, let X,Z be independent random variables with bounded second moments $E[X^{2}]<\infty$, $E[Z^{2}]<\infty$. Then, the expected Moreau envelope function $E\left[M_{\ell }\left(cX+Z;\lambda \right)\right]$ is differentiable with respect to both c and λ and the derivatives are given as follows:*

(A13) $$\begin{array}{cc} \frac{\partial }{\partial c}E\left[M_{\ell }\left(cX+Z;\lambda \right)\right] & =E\left[XM_{\ell ,1}^{\prime }\left(cX+Z;\lambda \right)\right], \end{array}$$

(A14) $$\begin{array}{cc} \frac{\partial }{\partial \lambda }E\left[M_{\ell }\left(cX+Z;\lambda \right)\right] & =E\left[M_{\ell ,2}^{\prime }\left(cX+Z;\lambda \right)\right]. \end{array}$$

**Proof.** The proof is an application of the Dominated Convergence Theorem (DCT). First, by Proposition A1(b), for every c∈R and any λ>0, the function $E[M_{\ell }\left(cX+Z;\lambda \right)]$ takes a finite value. Second, by Proposition A1(c), $M_{\ell }\left(cx+z;\lambda \right)$ is continuously differentiable with respect to both *c* and λ:

$$\begin{array}{cc} \frac{\partial }{\partial c}M_{\ell }\left(cX+Z;\lambda \right) & =XM_{\ell ,1}^{\prime }\left(cX+Z;\lambda \right)=X\frac{1}{\lambda }\left(cX+Z-{\mathrm{prox}}_{\ell }\left(cX+Z;\lambda \right)\right),\\ \frac{\partial }{\partial \lambda }M_{\ell }\left(cX+Z;\lambda \right) & =M_{\ell ,2}^{\prime }\left(cX+Z;\lambda \right)=-\frac{1}{2\lambda^{2}}{\left(cX+Z-{\mathrm{prox}}_{\ell }\left(cX+Z;\lambda \right)\right)}^{2}. \end{array}$$

From this, note that the Cauchy–Schwarz inequality gives

$$E\left[\frac{\partial }{\partial c}M_{\ell }\left(cX+Z;\lambda \right)\right]\leq \left(E\left[X^{2}\right]{)}^{1/2}\right){\left(E\left[\frac{1}{\lambda^{2}}\underset{:=A}{\underset{︸}{{\left(cX+Z-{\mathrm{prox}}_{\ell }\left(cX+Z;\lambda \right)\right)}^{2}}}\right]\right)}^{1/2},$$

Therefore, the remaining condition to check so that DCT can be applied is that the term $A/\lambda^{2}$ above is integrable. To begin with, we can easily bound *A* as: $A\leq 2{\left(cX+Z\right)}^{2}+2{\left({\mathrm{prox}}_{\ell }\left(cX+Z;\lambda \right)\right)}^{2}.$ Next, by non-expansiveness (Lipschitz property) of the proximal operator [52] (Prop. 12.19), we have that $|{\mathrm{prox}}_{\ell }\left(cX+Z;\lambda \right)\left|\leq |cX+Z|+\right|{\mathrm{prox}}_{\ell }\left(0;\lambda \right)|.$ Putting together, we find that

$$A\leq 6{\left(cX+Z\right)}^{2}+2|{\mathrm{prox}}_{\ell }\left(0;\lambda \right){|}^{2}\leq 12c^{2}X^{2}+12Z^{2}+2{\left|{\mathrm{prox}}_{\ell }\left(0;\lambda \right)\right|}^{2}.$$

We consider two cases. First, for fixed λ>0 and any compact interval I, we have that

$$E\underset{c\in I}{\sup}\left[A\right]\leq 12\left(\underset{c\in I}{\sup}c^{2}\right)E\left[X^{2}\right]+12E{\left[Z\right]}^{2}+2{\left|{\mathrm{prox}}_{\ell }\left(0;\lambda \right)\right|}^{2}<\infty .$$

Similarly, for fixed *c* and any compact interval J on the positive real line, we have that

$$E\underset{\lambda \in J}{\sup}\left[A/\lambda^{2}\right]\leq 12\underset{\lambda \in J}{\sup}\frac{c^{2}E\left[X^{2}\right]+E{\left[Z\right]}^{2}}{\lambda^{2}}+2\underset{\lambda \in J}{\sup}\frac{|{\mathrm{prox}}_{\ell }\left(0;\lambda \right){|}^{2}}{\lambda^{2}}<\infty ,$$

where we also used boundedness of the proximal operator (cf. Proposition A1(a)). This completes the proof. □

#### Appendix A.6.2. Strict Convexity

We study convexity properties of the *expected Moreau envelope function* $\Psi :R^{3}\to R$:

(A15) $$\begin{array}{c} \Psi \left(v\right):=\Psi \left(\alpha ,\mu ,\lambda \right):=E\left[M_{\ell }\left(\alpha X+\mu Z;\lambda \right)\right], \end{array}$$

for a lsc, proper, convex function *ℓ* and independent random variables *X* and *Z* with positive densities. Here, and onwards, we let $v\in R^{3}$ denote a triplet (α,μ,λ) and the expectation is over the randomness of *X* and *Z*. From Lemma A2, it is easy to see that Ψ(v) is convex. In this section, we prove a stronger claim:

“ If *ℓ* is strictly convex and does not attain its minimum at 0, then Ψ(v) is also strictly convex. ”

This is summarized in Proposition A5 below.

**Proposition** **A5.** (Strict Convexity). *Let ℓ:R→R be a function with the following properties: (i) it is proper strictly convex; and (ii) it is continuously differentiable and its derivative $\ell^{\prime }$ is such that $\ell^{\prime }\left(0\right)\neq 0$. Further, let X,Z be independent random variables with strictly positive densities. Then, the function $\Psi :R^{3}\to R$ in (A15) is jointly strictly convex in its arguments.*

**Proof.** Let $v_{i}=\left(\alpha_{i},\mu_{i},\lambda_{i}\right),i=1,2$, θ∈(0,1) and $\bar{\theta }=1-\theta$. Further, assume that $v_{1}\neq v_{2}$ and define the proximal operators

$$p_{i}\left(X,Z\right):={\mathrm{prox}}_{\ell }\left(\alpha_{i}X+\mu_{i}Z;\lambda_{i}\right)=\arg\min_{v}\frac{1}{2\lambda_{i}}{\left(\alpha_{i}X+\mu_{i}Z-v\right)}^{2}+\ell \left(v\right),$$

for i=1,2. Finally, denote $\lambda_{\theta }:=\theta \lambda_{1}+\bar{\theta }\lambda_{2},\alpha_{\theta }:=\theta \alpha_{1}+\bar{\theta }\alpha_{2}$ and $\mu_{\theta }:=\theta \mu_{1}+\bar{\theta }\mu_{2}$. With this notation, -4.6cm0cm

(A16) $$\begin{array}{cc} \; & \Psi (\theta v_{1}+\bar{\theta }v_{2})\\ & \;\leq E\left[\;\frac{1}{2\lambda_{\theta }}{\left(\alpha_{\theta }X+\mu_{\theta }Z-\left(\theta p_{1}\left(X,Z\right)+\bar{\theta }p_{2}\left(X,Z\right)\right)\right)}^{2}+\ell \left(\theta p_{1}\left(X,Z\right)+\theta p_{2}\left(X,Z\right)\right)\;\right]\\ & \;=E\left[\;H\left(\alpha_{\theta }X+\mu_{\theta }Z,\theta p_{1}\left(X,Z\right)+\theta p_{2}\left(X,Z\right),\lambda_{\theta }\right)+\ell \left(\theta p_{1}\left(X,Z\right)+\bar{\theta }p_{2}\left(X,Z\right)\right)\;\right]\\ & \;\leq E[\;\theta H\left(\alpha_{1}X+\mu_{1}Z,p_{1}\left(X,Z\right),\lambda_{1}\right)+\bar{\theta }H\left(\alpha_{2}X+\mu_{2}Z,p_{2}\left(X,Z\right),\lambda_{2}\right)\\ & \;+\ell \left(\theta p_{1}\left(X,Z\right)+\bar{\theta }p_{2}\left(X,Z\right)\right)\;]. \end{array}$$

The first inequality above follows by the definition of the Moreau envelope in (A1). The equality in the second line uses the definition of the function $H:R^{3}\to R$ in (A10). Finally, the last inequality follows from convexity of *H* as proved in Lemma A1. Continuing from (59), we may use convexity of *ℓ* to find that

(A17) $$\begin{array}{cc} \; & \Psi (\theta v_{1}+\bar{\theta }v_{2})\\ & \leq E[\;\theta H\left(\alpha_{1}X+\mu_{1}Z,\lambda_{1},p_{1}\left(X,Z\right)\right)+\bar{\theta }H\left(\alpha_{2}X+\mu_{2}Z,\lambda_{2},p_{2}\left(X,Z\right)\right)\\ & \;+\theta \ell \left(p_{1}\left(X,Z\right)\right)+\bar{\theta }\ell \left(p_{2}\left(X,Z\right)\right)]\\ & =\theta \Psi \left(v_{1}\right)+\bar{\theta }\Psi \left(v_{2}\right). \end{array}$$

This already proves convexity of (A15). In what follows, we argue that the inequality in (A17) is in fact strict under the assumption of the lemma. Specifically, in Lemma A3, we prove that, under the assumptions of the proposition, for $v_{1}\neq v_{2}$, it holds that

$$E\left[\ell \left(\theta p_{1}\left(X,Z\right)+\bar{\theta }p_{2}\left(X,Z\right)\right)\right]<\theta \;E\left[\ell \left(p_{1}\left(X,Z\right)\right)\right]+\bar{\theta }\;E\left[\ell \left(p_{2}\left(X,Z\right)\right)\right].$$

Using this in (A16) completes the proof of the proposition. The idea behind the proof of Lemma A3 is as follows. First, we use the fact that $v_{1}\neq v_{2}$ and $\ell^{\prime }\left(0\right)\neq 0$ to argue that there exists a non-zero measure set of $\left(x,z\right)\in R^{2}$ such that $p_{1}\left(x,z\right)\neq p_{2}\left(x,z\right)$. Then, the desired claim follows by *strict* convexity of *ℓ*. □

**Lemma** **A3.** *Let ℓ:R→R be a proper strictly convex function that is continuously differentiable with $\ell^{\prime }\left(0\right)\neq 0$. Further, assume independent continuous random variables X,Z with strictly positive densities. Fix arbitrary triplets $v_{i}=\left(\alpha_{i},\mu_{i},\lambda_{i}\right),i=1,2$ such that $v_{1}\neq v_{2}$. Further, denote*

(A18) $$\begin{array}{c} p_{i}\left(X,Z\right):={\mathrm{prox}}_{\ell }\left(\alpha_{i}X+\mu_{i}Z;\lambda_{i}\right),i=1,2. \end{array}$$

*Then, there exists a ball $S\subset R^{2}$ of nonzero measure, i.e., $P\left((X,Z)\in S\right)>0$, such that $p_{1}\left(x,z\right)\neq p_{2}\left(x,z\right)$, for all (x,z)∈S. Consequently, for any θ∈(0,1) and $\bar{\theta }=1-\theta$, the following strict inequality holds,*

(A19) $$\begin{array}{c} E\left[\ell \left(\theta p_{1}\left(X,Z\right)+\bar{\theta }p_{2}\left(X,Z\right)\right)\right]<\theta \;E\left[\ell \left(p_{1}\left(X,Z\right)\right)\right]+\bar{\theta }\;E\left[\ell \left(p_{2}\left(X,Z\right)\right)\right]. \end{array}$$

**Proof.** Note that (A19) holds trivially with “<" replaced by “≤" due to the convexity of *ℓ*. To prove that the inequality is strict, it suffices, by strict convexity of *ℓ*, that there exists subset $S\subset R^{2}$ that satisfies the following two properties:

1. $p_{1}\left(x,z\right)\neq p_{2}\left(x,z\right)$, for all (x,z)∈S.
2. $P\left((X,Z)\in S\right)>0$.

Consider the following function $f:R^{2}\to R$:

(A20) $$\begin{array}{c} f\left(x,z\right):=p_{1}\left(x,z\right)-p_{2}\left(x,z\right). \end{array}$$

By Lemma A4, there exists $\left(x_{0},z_{0}\right)$ such that

(A21) $$\begin{array}{c} f(x_{0},z_{0})\neq 0. \end{array}$$

Moreover, by continuity of the proximal operator (cf. Proposition A1(a)), it follows that *f* is continuous. From this and (A21), we conclude that for sufficiently small ζ>0 there exists a ζ-ball S centered at $\left(x_{0},z_{0}\right)$, such that property 1 holds. Property 2 is also guaranteed to hold for S, since both X,Z have strictly positive densities and are independent. □

**Lemma** **A4.** *Let ℓ:R→R be a proper, convex function. Further, assume that ℓ:R→R is continuously differentiable and $\ell^{\prime }\left(0\right)\neq 0$. Let $\alpha_{1},\alpha_{2}>0$, $\lambda_{1},\lambda_{2}>0$. Then, the following statement is true*
*-4.6cm0cm*

(A22) $$\begin{array}{c} \;\left(\alpha_{1},\mu_{1},\lambda_{1}\right)\neq \left(\alpha_{2},\mu_{2},\lambda_{2}\right)\to \exists \left(x,z\right)\in R^{2}:{\mathrm{prox}}_{\ell }\left(\alpha_{1}x+\mu_{1}z;\lambda_{1}\right)\neq {\mathrm{prox}}_{\ell }\left(\alpha_{2}x+\mu_{2}z;\lambda_{2}\right). \end{array}$$

**Proof.** We prove the claim by contradiction, but first, let us set up some useful notation. Let $v\in R^{3}$ denote triplets (α,μ,λ) and further define

$$p_{\alpha ,\mu ,\lambda }\left(x,z\right):={\mathrm{prox}}_{\ell }\left(\alpha x+\mu z;\lambda \right),$$

and

$$L_{\alpha ,\mu ,\lambda }\left(x,z\right):=\ell^{\prime }\left({\mathrm{prox}}_{\ell }\left(\alpha x+\mu z;\lambda \right)\right).$$

By Proposition A1, the following is true:

(A23) $$\begin{array}{c} L_{\alpha ,\mu ,\lambda }\left(x,z\right)=\frac{1}{\lambda }\left(\alpha x+\mu z-p_{\alpha ,\mu ,\lambda }\left(x,z\right)\right). \end{array}$$

For the sake of contradiction, assume that the claim of the lemma is false. Then,

(A24) $$\begin{array}{c} p_{\alpha_{1},\mu_{1},\lambda_{1}}\left(x,z\right)=p_{\alpha_{2},\mu_{2},\lambda_{2}}\left(x,z\right),\;\forall \left(x,z\right)\in R^{2}. \end{array}$$

From this, it also holds that

(A25) $$\begin{array}{c} L_{\alpha_{1},\mu_{1},\lambda_{1}}\left(x,z\right)=L_{\alpha_{2},\mu_{2},\lambda_{2}}\left(x,z\right),\;\forall \left(x,z\right)\in R^{2}. \end{array}$$

Recalling (A23) and applying (A24), we derive the following from (A25):

(A26) $$\begin{array}{c} \left(\lambda_{2}-\lambda_{1}\right)p_{\alpha_{1},\mu_{1},\lambda_{1}}\left(x,z\right)=\left(\lambda_{2}\alpha_{1}-\lambda_{1}\alpha_{2}\right)x+\left(\lambda_{2}\mu_{1}-\lambda_{1}\mu_{2}\right)z,\;\forall \left(x,z\right)\in R^{2}. \end{array}$$

We consider the following two cases separately. Case 1: $\lambda_{1}=\lambda_{2}$: Since $v_{1}\neq v_{2}$, it holds that

(A27) $$\begin{array}{c} \exists \left(x,z\right)\in R^{2}:\;\alpha_{1}x+\mu_{1}z\neq \alpha_{2}x+\mu_{2}z. \end{array}$$

However, from (A26) we have that $\left(\alpha_{1}-\alpha_{2}\right)x+\left(\mu_{1}-\mu_{2}\right)z=0$ for all $\left(x,z\right)\in R^{2}$. This contradicts (A27) and completes the proof for this case. Case 2: $\lambda_{1}\neq \lambda_{2}$: Continuing from (A26), we can compute that for all $\left(x,z\right)\in R^{2}$

(A28) $$\begin{array}{cc} \ell^{\prime }\left(p_{\alpha_{1},\mu_{1},\lambda_{1}}\left(x,z\right)\right) & =\frac{1}{\lambda_{1}}\left(\alpha_{1}x+\mu_{1}z-p_{\alpha_{1},\mu_{1},\lambda_{1}}\left(x,z\right)\right)\\ & =\frac{\alpha_{2}-\alpha_{1}}{\lambda_{2}-\lambda_{1}}x+\frac{\mu_{2}-\mu_{1}}{\lambda_{2}-\lambda_{1}}z. \end{array}$$

By replacing $p_{\alpha_{1},\mu_{1},\lambda_{1}}\left(x,z\right)$ from (A26), we derive that:

(A29) $$\begin{array}{c} \ell^{\prime }\left(\varepsilon_{1}x+\varepsilon_{2}z\right)=\varepsilon_{3}x+\varepsilon_{4}z,\;\forall \left(x,z\right)\in R^{2}, \end{array}$$

where

$$\begin{array}{cc} \varepsilon_{1} & =\frac{\lambda_{2}\alpha_{1}-\lambda_{1}\alpha_{2}}{\lambda_{2}-\lambda_{1}},\;\varepsilon_{2}=\frac{\lambda_{2}\mu_{1}-\lambda_{1}\mu_{2}}{\lambda_{2}-\lambda_{1}},\\ \varepsilon_{3} & =\frac{\alpha_{2}-\alpha_{1}}{\lambda_{2}-\lambda_{1}},\;\;\;\varepsilon_{4}=\frac{\mu_{2}-\mu_{1}}{\lambda_{2}-\lambda_{1}}. \end{array}$$

By replacing x=z=0 in (A29), we find that $\ell^{\prime }\left(0\right)=0$. This contradicts the assumption of the lemma and completes the proof. □

#### Appendix A.6.3. Strict Concavity

In this section, we study the following variant $\Gamma :R_{+}\to R$ of the expected Moreau envelope:

(A30) $$\begin{array}{c} \Gamma \left(\gamma \right):=E\left[M_{\ell }\left(X;1/\gamma \right)\right], \end{array}$$

for a lower semi-continuous, proper, convex function *ℓ* and continuous random variable *X*. The expectation above is over the randomness of *X*. In Appendix B.4, we show that the function Γ is concave in γ. Here, we prove the following statement regarding *strict*-concavity of Γ:

“ If *ℓ* is convex, continuously differentiable and $\ell^{\prime }\left(0\right)\neq 0$, then Γ is *strictly* concave. ”

This is summarized in Proposition A6 below.

**Proposition** **A6.** (Strict concavity). *Let ℓ:R→R be a convex, continuously differentiable function for which $\ell^{\prime }\left(0\right)\neq 0$. Further, let X be a continuous random variable in R with strictly positive density in the real line. Then, the function Γ in (A23) is strictly concave in $R_{+}$.*

**Proof.** Before everything, we introduce the following convenient notation:

$${\tilde{\Gamma }}_{x}\left(\gamma \right):=M_{\ell }\left(x;1/\gamma \right)\;\mathrm{and}\;p_{\gamma }^{x}:={\mathrm{prox}}_{\ell }\left(x;1/\gamma \right).$$

Note from Proposition A1 that ${\tilde{\Gamma }}_{x}$ is differentiable with derivative

(A31) $$\begin{array}{c} {\tilde{\Gamma }}_{x}^{\prime }\left(\gamma \right)=\frac{1}{2}{\left(x-{\mathrm{prox}}_{\ell }\left(x;1/\gamma \right)\right)}^{2}. \end{array}$$

We proceed in two steps as follows. First, for fixed x∈R and $\gamma_{2}>\gamma_{1}$, we prove in Lemma A5 that

(A32) $$\begin{array}{c} {\left(x-p_{\gamma_{2}}^{x}\right)}^{2}-{\left(x-p_{\gamma_{1}}^{x}\right)}^{2}\leq -\frac{\gamma_{1}}{\gamma_{2}-\gamma_{1}}{\left(p_{\gamma_{1}}^{x}-p_{\gamma_{2}}^{x}\right)}^{2}, \end{array}$$

This shows that for all x∈R

(A33) $$\begin{array}{c} {\tilde{\Gamma }}_{x}^{\prime }\left(\gamma_{2}\right)-{\tilde{\Gamma }}_{x}^{\prime }\left(\gamma_{1}\right)\leq 0. \end{array}$$

Second, we use Lemma A3 to argue that the inequality is in fact strict for all x∈S where S⊂R and P(X∈S)>0. To be concrete, apply Lemma A3 for $v_{i}=\left(1,0,1/\gamma_{i}\right),i=1,2$. Notice that all the assumptions of the lemma are satisfied, hence there exists interval S⊂R for which P(X∈S)>0 and

$$p_{\gamma_{1}}^{x}\neq p_{\gamma_{2}}^{x}\Rightarrow {\left(p_{\gamma_{1}}^{x}-p_{\gamma_{2}}^{x}\right)}^{2}>0,\;\forall x\in S.$$

Hence, from (A32), it follows that

$${\left(x-p_{\gamma_{2}}^{x}\right)}^{2}-{\left(x-p_{\gamma_{1}}^{x}\right)}^{2}<0,\;\forall x\in S.$$

From this, and (A31) we conclude that

(A34) $$\begin{array}{c} {\tilde{\Gamma }}_{x}^{\prime }\left(\gamma_{2}\right)-{\tilde{\Gamma }}_{x}^{\prime }\left(\gamma_{1}\right)<0,\;\forall x\in S. \end{array}$$

Thus, from (A33) and (A34), as well as the facts that $\Gamma \left(\gamma \right)=E\left[{\tilde{\Gamma }}_{X}\left(\gamma \right)\right]$ and P(X∈S)>0, we conclude that Γ is strictly concave in $R_{+}$. □

**Lemma** **A5.** *Let ℓ:R→R be a convex, continuously differentiable function. Fix x∈R and denote $p_{\gamma }:={\mathrm{prox}}_{\ell }\left(x;1/\gamma \right)$. Then, for any $\gamma ,\tilde{\gamma }>0$, it holds that*

(A35) $$\begin{array}{c} \left(\tilde{\gamma }-\gamma \right)\left(p_{\tilde{\gamma }}-p_{\gamma }\right)\left(p_{\gamma }-x\right)+\tilde{\gamma }{\left(p_{\tilde{\gamma }}-p_{\gamma }\right)}^{2}\leq 0. \end{array}$$

*Moreover, for $\gamma_{2}>\gamma_{1}$, the following statement is true:*

(A36) $$\begin{array}{c} {\left(x-p_{\gamma_{2}}\right)}^{2}-{\left(x-p_{\gamma_{1}}\right)}^{2}\leq -\frac{\gamma_{1}}{\gamma_{2}-\gamma_{1}}{\left(p_{\gamma_{1}}-p_{\gamma_{2}}\right)}^{2}. \end{array}$$

**Proof.** First, we prove (A35). Then, we use it to prove (A36). Proof of (A35): Consider function g:R→R defined as follows $g\left(p\right)=\frac{\tilde{\gamma }}{2}{\left(x-p\right)}^{2}+\ell \left(p\right)$. By assumption, *g* is differentiable with derivative $g^{\prime }\left(p\right)=\tilde{\gamma }\left(p-x\right)+\ell^{\prime }\left(p\right)$. Moreover, *g* is $\gamma_{2}$-strongly convex. Finally, by optimality of the proximal operator (cf. Proposition A1), it holds that $\gamma \left(x-p_{\gamma }\right)=\ell^{\prime }\left(p_{\gamma }\right)$ and $\tilde{\gamma }\left(x-p_{\tilde{\gamma }}\right)=\ell^{\prime }\left(p_{\tilde{\gamma }}\right)$. Using these, it can be computed that $g^{\prime }\left(p_{\tilde{\gamma }}\right)=0$ and $g^{\prime }\left(p_{\gamma }\right)=\left(\tilde{\gamma }-\gamma \right)\left(p_{\gamma }-x\right)$. In the following inequalities, we combine all the aforementioned properties of the function *g* to find that

$$\begin{array}{cc} g(p_{\gamma }) & \geq g\left(p_{\tilde{\gamma }}\right)+\frac{\tilde{\gamma }}{2}{\left(p_{\gamma }-p_{\tilde{\gamma }}\right)}^{2}\geq g\left(p_{\gamma }\right)+\left(\tilde{\gamma }-\gamma \right)\left(p_{\gamma }-x\right)\left(p_{\tilde{\gamma }}-p_{\gamma }\right)+\tilde{\gamma }{\left(p_{\gamma }-p_{\tilde{\gamma }}\right)}^{2}. \end{array}$$

This leads to the desired statement and completes the proof of (A35). Proof of (A36): We fix $\gamma_{2}>\gamma_{1}$ and apply (A35) two times as follows. First, applying (A35) for $\left(\tilde{\gamma },\gamma \right)=\left(\gamma_{2},\gamma_{1}\right)$ and using the fact that $\gamma_{2}>\gamma_{1}$, we find that

(A37) $$\begin{array}{c} \left(p_{\gamma_{2}}-p_{\gamma_{1}}\right)\left(p_{\gamma_{1}}-x\right)\leq -\frac{\gamma_{2}}{\gamma_{2}-\gamma_{1}}{\left(p_{\gamma_{2}}-p_{\gamma_{1}}\right)}^{2}. \end{array}$$

Second, applying (A35) for $\left(\tilde{\gamma },\gamma \right)=\left(\gamma_{1},\gamma_{2}\right)$ and using again the fact that $\gamma_{2}>\gamma_{1}$, we find that

(A38) $$\begin{array}{cc} & \left(\gamma_{1}-\gamma_{2}\right)\left(p_{\gamma_{1}}-p_{\gamma_{2}}\right)\left(p_{\gamma_{2}}-x\right)+\gamma_{1}{\left(p_{\gamma_{1}}-p_{\gamma_{2}}\right)}^{2}\leq 0\\ \Rightarrow \; & \left(p_{\gamma_{2}}-p_{\gamma_{1}}\right)\left(p_{\gamma_{2}}-x\right)\leq -\frac{\gamma_{1}}{\gamma_{2}-\gamma_{1}}{\left(p_{\gamma_{1}}-p_{\gamma_{2}}\right)}^{2}. \end{array}$$

Adding (A37) and (A38), we show the desired property as follows:

$$\left(p_{\gamma_{2}}-p_{\gamma_{1}}\right)\left(p_{\gamma_{2}}-x\right)+\left(p_{\gamma_{2}}-p_{\gamma_{1}}\right)\left(p_{\gamma_{1}}-x\right)\leq -\frac{\gamma_{2}+\gamma_{1}}{\gamma_{2}-\gamma_{1}}{\left(p_{\gamma_{1}}-p_{\gamma_{2}}\right)}^{2}.$$

□

#### Appendix A.6.4. Summary of Properties of (uid135)

**Proposition** **A7.** *Let ℓ:R→R be a lsc, proper, convex function. Let $G,S\overset{\mathrm{iid}}{∼}N\left(0,1\right)$ and function f:R→{±1} such that the random variable YS=f(S)S has a continuous strictly positive density on the real line. Then, the following properties are true for the expected Moreau envelope function*

(A39) $$\begin{array}{c} \Omega :\left(\alpha >0,\mu ,\tau >0,\gamma >0\right)\mapsto E\left[M_{\ell }\left(\alpha G+\mu SY;\tau /\gamma \right)\right]: \end{array}$$

(a) *The function Ω is differentiable and its derivatives are given as follows:*
  $$\begin{array}{cc} \frac{\partial }{\partial \alpha }\Omega \left(\alpha ,\mu ,\tau ,\gamma \right) & =E\left[G\;M_{\ell ,1}^{\prime }\left(\alpha G+\mu SY;\tau /\gamma \right)\right],\\ \frac{\partial }{\partial \mu }\Omega \left(\alpha ,\mu ,\tau ,\gamma \right) & =E\left[SY\;M_{\ell ,1}^{\prime }\left(\alpha G+\mu SY;\tau /\gamma \right)\right],\\ \frac{\partial }{\partial \tau }\Omega \left(\alpha ,\mu ,\tau ,\gamma \right) & =\frac{1}{\gamma }E\left[M_{\ell ,2}^{\prime }\left(\alpha G+\mu SY;\tau /\gamma \right)\right],\\ \frac{\partial }{\partial \gamma }\Omega \left(\alpha ,\mu ,\tau ,\gamma \right) & =-\frac{\tau }{\gamma^{2}}E\left[M_{\ell ,2}^{\prime }\left(\alpha G+\mu SY;\tau /\gamma \right)\right]. \end{array}$$
(b) The function Ω is jointly convex (α,μ,τ) and concave on γ.
(c) The function Ω is increasing in α.
  For the statements below, further assume that ℓ is strictly convex and continuously differentiable with $\ell^{\prime }\left(0\right)\neq 0$.
(d) *The function Ω is strictly convex in (α,μ,τ) and strictly concave in λ.*
(e) The function Ω is strictly increasing in α.

**Proof.** Statements (a), (b) and (d) follow directly by Propositions A4–A6. It remains to prove Statements (c) and (e). Let $\alpha_{2}>\alpha_{1}$. Then, there exist independent copies $G^{\prime },G^{\prime \prime }$ of *G* and $\tilde{\alpha }>0$ such that $\alpha_{2}G=\alpha_{1}G^{\prime }+\tilde{\alpha }G^{\prime \prime }$. Hence, we have the following chain of inequalities:

$$\begin{array}{cc} \Omega (\alpha_{2},\mu ,\tau ,\gamma ) & =E\left[M_{\ell }\left(\alpha_{1}G^{\prime }+\tilde{\alpha }G^{\prime \prime }+\mu SY;\tau /\gamma \right)\right]\geq E\left[M_{\ell }\left(\alpha_{1}G^{\prime }+\tilde{\alpha }E\left[G^{\prime \prime }\right]+\mu SY;\tau /\gamma \right)\right]\\ \; & =E\left[M_{\ell }\left(\alpha_{1}G^{\prime }+\mu SY;\tau /\gamma \right)\right]=\Omega \left(\alpha_{1},\mu ,\tau ,\gamma \right), \end{array}$$

where the inequality follows from Jensen and convexity of Ω with respect to α (see Statement (b) of the Proposition). This proves Statement (c). For Statement (e), note that the inequality is strict provided that Ω is strictly convex (see Statement (d) of the Proposition). □

## Appendix B. Proof of Theorem 1

In this section, we provide a proof sketch of Theorem 1. The main technical tool that facilitates our analysis is the convex Gaussian min-max theorem (CGMT), which is an extension of Gordon’s Gaussian min-max inequality (GMT). We introduce the necessary background on the CGMT in Appendix B.1.

The CGMT has been mostly applied to linear measurements [9,10,13,15,19]. The simple, yet central idea, which allows for this extension, is a certain projection trick inspired by Plan and Vershynin [40]. Here, we apply a similar trick, but, in our setting, we recognize that it suffices to simply rotate $x_{0}$ to align with the first basis vector. The simple rotation decouples the measurements $y_{i}$ from the last n−1 coordinates of the measurement vectors $a_{i}$ (see Appendix B.2). While this is sufficient for LS in [43], to study more general loss functions, we further need to combine this with a duality argument similar to that in [13]. Second, while the steps that bring the ERM minimization to the form of a PO (see (A48)) bear the aforementioned similarities to those in [13,43], the resulting AO is different from the one studied in previous works. Hence, the mathematical derivations in Appendix B.3 and Appendix B.4 are different. This also leads to a different system of equations characterizing the statistical behavior of ERM. Finally, in Appendix B.5, we prove uniqueness of the solution of this system of equations using the properties of the expected Moreau envelope function studied in Appendix A.6.

### Appendix B.1. Technical Tool: CGMT

#### Appendix B.1.1. Gordon’s Min-Max Theorem (GMT)

The Gordon’s Gaussian comparison inequality [59] compares the min-max value of two doubly indexed Gaussian processes based on how their autocorrelation functions compare. The inequality is quite general (see [59]), but for our purposes we only need its application to the following two Gaussian processes:

(A40a) $$\begin{array}{cc} X_{w,u} & :=u^{T}Gw+\psi \left(w,u\right), \end{array}$$

(A40b) $$\begin{array}{cc} Y_{w,u} & :={∥w∥}_{2}g^{T}u+{∥u∥}_{2}Tw+\psi \left(w,u\right), \end{array}$$

where $G\in R^{m\times n}$, $g\in R^{m}$, $R^{n}$, they all have entries iid Gaussian; the sets $S_{w}\subset R^{n}$ and $S_{u}\subset R^{m}$ are compact; and $\psi :R^{n}\times R^{m}\to R$. For these two processes, define the following (random) min-max optimization programs, which we refer to as the *primary optimization* (PO) problem and the *auxiliary optimization* (AO).

(A41a) $$\begin{array}{cc} \tilde{\Phi }\left(G\right) & =\min_{w\in S_{w}}\max_{u\in S_{u}}X_{w,u}, \end{array}$$

(A41b) $$\begin{array}{cc} \phi (g, & =\min_{w\in S_{w}}\max_{u\in S_{u}}Y_{w,u}. \end{array}$$

According to Gordon’s comparison inequality (To be precise, the formulation in (A42), which is due to [13], is slightly different from the original statement in Gordon’s paper (see [13] for details).), for any c∈R, it holds:

(A42) $$P\left(\tilde{\Phi }\left(G\right)<c\right)\leq 2P\left(\phi (g,<c\right).$$

In other words, a high-probability lower bound on the AO is a high-probability lower bound on the PO. The premise is that it is often much simpler to lower bound the AO rather than the PO. To be precise, (A42) is a slight reformulation of Gordon’s original result proved in [13].

#### Appendix B.1.2. Convex Gaussian Min-Max Theorem (CGMT)

The proof of Theorem 1 builds on the CGMT [13]. For ease of reference, we summarize here the essential ideas of the framework following the presentation in [15] (please see [15] (Section 6) for the formal statement of the theorem and further details). The CGMT is an extension of the GMT and it asserts that the AO in (41b) can be used to tightly infer properties of the original (PO) in (41a), including the optimal cost and the optimal solution. According to the CGMT [15] (Theorem 6.1), if the sets $S_{w}$ and $S_{u}$ are convex and ψ is continuous *convex-concave* on $S_{w}\times S_{u}$, then, for any ν∈R and t>0, it holds that

(A43) $$P\left(\;|\tilde{\Phi }\left(G\right)-\nu |>t\right)\leq 2P\left(\;|\phi (g,-\nu |>t\right).$$

In words, concentration of the optimal cost of the AO problem around μ implies concentration of the optimal cost of the corresponding PO problem around the same value μ. Moreover, starting from (A43) and under strict convexity conditions, the CGMT shows that concentration of the optimal solution of the AO problem implies concentration of the optimal solution of the PO to the same value. For example, if minimizers of (A41b) satisfy $∥w^{*}{(g,∥}_{2}\to \zeta^{*}$ for some $\zeta^{*}>0$, then the same holds true for the minimizers of (A41a): ${∥w^{*}\left(G\right)∥}_{2}\to \zeta^{*}$ [15] ([Theorem 6.1(iii)). Thus, one can analyze the AO to infer corresponding properties of the PO, the premise being of course that the former is simpler to handle than the latter.

### Appendix B.2. Applying the CGMT to ERM for Binary Classification

In this section, we show how to apply the CGMT to (3). For convenience, we drop the subscript *ℓ* from ${\hat{x}}_{\ell }$ and simply write

(A44) $$\hat{x}=\arg\min_{x}\frac{1}{m}\sum_{i=1}^{m}\ell \left(y_{i}a_{i}^{T}x\right),$$

where the measurements $y_{i},i\in \left[m\right]$ follow (1). By rotational invariance of the Gaussian distribution of the measurement vectors $a_{i},i\in \left[m\right]$, we assume without loss of generality that $x_{0}={\left[1,0,\ldots ,0\right]}^{T}$. We can rewrite (A44) as a constrained optimization problem by introducing *n* variables $u_{i}$ as follows:

$$\hat{x}=\arg\min_{x,u}\frac{1}{m}\sum_{i=1}^{m}\ell \left(u_{i}\right)\mathrm{subject}\;\mathrm{to}\;u_{i}=y_{i}a_{i}^{T}x,i\in \left[n\right].$$

This problem is now equivalent to the following min-max formulation:

(A45) $$\begin{array}{c} \min_{u,x}\max_{\beta }\frac{1}{m}\sum_{i=1}^{m}\ell \left(u_{i}\right)+\frac{1}{m}\sum_{i=1}^{m}\beta_{i}u_{i}-\frac{1}{m}\sum_{i=1}^{m}\beta_{i}y_{i}a_{i}^{T}x. \end{array}$$

Now, let us define

$$a_{i}=\left[s_{i};{\tilde{a}}_{i}\right],i\in \left[m\right]\;\;\mathrm{and}\;\;x=\left[x_{1};\tilde{x}\right],$$

such that $s_{i}$ and $x_{1}$ are the first entries of $a_{i}$ and x, respectively. Note that in this new notation (1) becomes:

(A46) $$\begin{array}{c} y_{i}=f\left(s_{i}\right), \end{array}$$

and

(A47) $$\begin{array}{c} \mathrm{corr}\left(\;\hat{x}\;;\;x_{0}\;\right)=\frac{{\hat{x}}_{1}}{\sqrt{{\hat{x}}_{1}^{2}+{∥\tilde{\hat{x}}∥}_{2}^{2}}}, \end{array}$$

where we decompose $\hat{x}=\left[{\hat{x}}_{1};\tilde{\hat{x}}\right]$. In addition, (A45) is written as

$$\min_{u,x}\max_{\beta }\frac{1}{m}\sum_{i=1}^{m}\ell \left(u_{i}\right)+\frac{1}{m}\sum_{i=1}^{m}\beta_{i}u_{i}+\frac{1}{m}\sum_{i=1}^{m}\beta_{i}y_{i}{\tilde{a}}_{i}^{T}\tilde{x}-\frac{1}{m}\sum_{i=1}^{m}\beta_{i}y_{i}s_{i}x_{1}$$

or, in matrix form, as

(A48) $$\min_{u,x}\max_{\beta }\frac{1}{m}\beta^{T}D_{y}\tilde{A}\tilde{x}+\frac{1}{m}x_{1}\beta^{T}D_{y}s+\frac{1}{m}\beta^{T}u+\frac{1}{m}\sum_{i=1}^{m}\ell \left(u_{i}\right).$$

where $D_{y}:=\mathrm{diag}\left(y_{1},y_{2},\ldots ,y_{m}\right)$ is a diagonal matrix with $y_{1},y_{2},\ldots y_{m}$ on the diagonal, $s={\left[s_{1},\ldots ,s_{m}\right]}^{T}$ and $\tilde{A}$ is an m×(n−1) matrix with rows ${\tilde{a}}_{i}^{T},i\in \left[m\right]$.

In (A48), we recognize that the first term has the bilinear form required by the GMT in (A41a). The rest of the terms form the function ψ in (A41a): they are independent of $\tilde{A}$ and convex-concave as desired by the CGMT. Therefore, we express (A44) in the desired form of a PO and for the rest of the proof we analyze the probabilistically equivalent AO problem. In view of (A41b), this is given as follows,

(A49) $$\min_{u,x}\max_{\beta }\frac{1}{m}{∥\tilde{x}∥}_{2}g^{T}D_{y}\beta +\frac{1}{m}{∥D_{y}\beta ∥}_{2}h^{T}\tilde{x}-\frac{1}{m}x_{1}\beta^{T}D_{y}s+\frac{1}{m}\beta^{T}u+\frac{1}{m}\sum_{i=1}^{m}\ell \left(u_{i}\right)\;,$$

where as in (A41b) $g∼N(0,I_{m})$ and $h∼N(0,I_{n-1})$.

### Appendix B.3. Analysis of the Auxiliary Optimization

Here, we show how to analyze the AO in (A49). To begin with, note that $y_{i}\in \left\{\pm 1\right\}$, therefore $D_{y}g∼N\left(0,I_{m}\right)$ and ${∥D_{y}\beta ∥}_{2}={∥\beta ∥}_{2}$. In addition, let us denote the first entry $x_{1}$ of x as

$$\mu :=x_{1}.$$

The first step is to optimize over the direction of $\tilde{x}$. For this, we express the AO as: -4.6cm0cm

(A50) $$\;\min_{u,\mu ,\alpha \geq 0}\min_{∥\tilde{x}{∥}_{2}=\alpha }\max_{\beta }\frac{1}{m}{∥\tilde{x}∥}_{2}g^{T}D_{y}\beta +\frac{1}{m}{∥D_{y}\beta ∥}_{2}h^{T}\tilde{x}-\frac{1}{m}\mu \beta^{T}D_{y}s+\frac{1}{m}\beta^{T}u+\frac{1}{m}\sum_{i=1}^{m}\ell \left(u_{i}\right)\;,$$

Now, denote ${\tilde{x}}_{☆}{=-\alpha ∥}_{2}$ and observe that for every β the objective above is minimized (with respect to to $\tilde{x}$) at ${\tilde{x}}_{☆}$. Thus, it follows by [23] (Lem. 8) that (A50) simplifies to

(A51) $$\min_{\alpha \geq 0,\mu ,u}\max_{\beta }\frac{1}{m}\alpha g^{T}\beta -\frac{\alpha }{m}{∥\beta ∥}_{2}{∥h∥}_{2}-\frac{1}{m}\mu s^{T}D_{y}\beta +\frac{1}{m}\beta^{T}u+\frac{1}{m}\sum_{i=1}^{m}\ell \left(u_{i}\right).$$

Next, let $\gamma :=\frac{{∥\beta ∥}_{2}}{\sqrt{m}}$ and optimize over the direction of β to yield

(A52) $$\min_{\alpha \geq 0,u,\mu }\max_{\gamma \geq 0}\frac{\gamma }{\sqrt{m}}{∥\alpha g-\mu D_{y}s+u∥}_{2}-\frac{\alpha }{\sqrt{m}}\gamma {∥h∥}_{2}+\frac{1}{m}\sum_{i=1}^{m}\ell \left(u_{i}\right).$$

To continue, we utilize the fact that for all x∈R, $\min_{\tau >0}\frac{\tau }{2}+\frac{x^{2}}{2\tau m}=\frac{x}{\sqrt{m}}$. Hence,

$$\begin{array}{c} \frac{\gamma }{\sqrt{m}}{∥\alpha g-\mu D_{y}s+u∥}_{2}=\min_{\tau >0}\frac{\gamma \tau }{2}+\frac{\gamma }{2\tau m}{∥-\alpha g+\mu D_{y}s-u∥}_{2}^{2}\;. \end{array}$$

With this trick, the optimization over u becomes separable over its coordinates $u_{i},i\in \left[m\right]$. By inserting this in (A42), we have

$$\min_{\alpha \geq 0,u,\mu }\max_{\gamma \geq 0}\min_{\tau >0}\frac{\gamma \tau }{2}-\frac{\alpha }{\sqrt{m}}\gamma {∥h∥}_{2}+\frac{\gamma }{2\tau m}\sum_{i=1}^{m}{\left(-\alpha g_{i}+\mu y_{i}s_{i}-u_{i}\right)}^{2}+\frac{1}{m}\sum_{i=1}^{m}\ell \left(u_{i}\right),$$

Now, we show that the objective function above is convex-concave. Clearly, the function is linear (thus, concave in γ). Moreover, from Lemma A1, the function $\frac{1}{2\tau }{\left(\alpha g_{i}+\mu y_{i}s_{i}-u_{i}\right)}^{2}$ is jointly convex in $\left(\alpha ,\mu ,u_{i},\tau \right)$. The rest of the terms are clearly convex and this completes the argument. Hence, with a permissible change in the order of min-max, we arrive at the following convenient form (Here, we skip certain technical details in this argument regarding boundedness of the constraint sets in (A49). While they are not trivial, they can be handled with the same techniques used in [15,60].):

(A53) $$\min_{\mu ,\alpha \geq 0,\tau >0}\max_{\gamma \geq 0}\frac{\gamma \tau }{2}-\frac{\alpha }{\sqrt{m}}\gamma {∥h∥}_{2}+\frac{1}{m}\sum_{i=1}^{m}M_{\ell }\left(-\alpha g_{i}+\mu s_{i}y_{i};\frac{\tau }{\gamma }\right),$$

where recall the definition of the Moreau envelope in (A1). As to now, we have reduced the AO into a random min-max optimization over only four scalar variables in (A53). For fixed μ,α,τ,γ, direct application of the weak law of large numbers shows that the objective function of (A53) converges in probability to the following as m,n→∞ and $\frac{m}{n}=\delta$:

$$\gamma \frac{\tau }{2}-\frac{\alpha \gamma }{\sqrt{\delta }}+E\left[M_{\ell }\left(\alpha G+\mu YS;\frac{\tau }{\gamma }\right)\right],$$

where G,S∼N(0,1) and Y∼f(S) (in view of (A46)). Based on that, it can be shown (similar arguments are developed in [15,60]) that the random optimizers $\alpha_{n}$ and $\mu_{n}$ of (A53) converge to the deterministic optimizers α and μ of the following (deterministic) optimization problem (whenever these are bounded as the statement of the theorem requires):

(A54) $$\min_{\alpha \geq 0,\mu ,\tau >0}\max_{\gamma \geq 0}\gamma \frac{\tau }{2}-\frac{\alpha \gamma }{\sqrt{\delta }}+E\left[M_{\ell }\left(\alpha G+\mu YS;\frac{\tau }{\gamma }\right)\right].$$

At this point, recall that α represents the norm of $\tilde{x}$ and μ the value of $x_{1}$. Thus, in view of (i) (A47); (ii) the equivalence between the PO and the AO; and (iii) our derivations thus far, we have that with probability approaching 1,

$$\begin{array}{c} \lim_{n\to +\infty }\mathrm{corr}\left(\;\hat{x}\;;\;x_{0}\;\right)=\frac{\mu }{\sqrt{\mu^{2}+\alpha^{2}}}, \end{array}$$

where μ and α are the minimizers in (A54). The three equations in (8) are derived by the first-order optimality conditions of the optimization in (A54). We show this next.

### Appendix B.4. Convex-Concavity and First-Order Optimality Conditions

First, we prove that the objective function in (A54) is convex–concave. For convenience define the function $F:R^{4}\to R$ as follows

(A55) $$\begin{array}{c} F\left(\alpha ,\mu ,\tau ,\gamma \right):=\frac{\gamma \tau }{2}-\frac{\alpha \gamma }{\sqrt{\delta }}+E\left[M_{\ell }\left(\alpha G+\mu YS;\frac{\tau }{\gamma }\right)\right]. \end{array}$$

Based on Lemma A2, it immediately follows that, if *ℓ* is convex, *F* is jointly convex in (α,μ,τ). To prove concavity of *F* based on γ, it suffices to show that $M_{\ell }\left(x;1/\gamma \right)$ is concave in γ for all x∈R. To show this, we note that

$$\begin{array}{c} M_{\ell }\left(x;1/\gamma \right)=\min_{u}\frac{\gamma }{2}{\left(x-u\right)}^{2}+\ell \left(u\right), \end{array}$$

which is the point-wise minimum of linear functions of γ. Thus, using Proposition A3(b), we conclude that $M_{\ell }\left(x;1/\gamma \right)$ is concave in γ. This completes the proof of convex-concavity of the function *F* in (A55) when *ℓ* is convex. By direct differentiation and applying Proposition A7(a), the first-order optimality conditions of the min–max optimization in (A54) are as follows:

(A56a) $$\begin{array}{cc} E\left[SY\cdot M_{\ell ,1}^{\prime }\left(\alpha G+\mu SY;\frac{\tau }{\gamma }\right)\right] & =0, \end{array}$$

(A56b) $$\begin{array}{cc} E\left[G\cdot M_{\ell ,1}^{\prime }\left(\alpha G+\mu SY;\frac{\tau }{\gamma }\right)\right] & =\frac{\gamma }{\sqrt{\delta }}, \end{array}$$

(A56c) $$\begin{array}{cc} \frac{\gamma }{2}+\frac{1}{\gamma }E\left[M_{\ell ,2}^{\prime }\left(\alpha G+\mu SY;\frac{\tau }{\gamma }\right)\right] & =0, \end{array}$$

(A56d) $$\begin{array}{cc} -\frac{\alpha }{\sqrt{\delta }}-\frac{\tau }{\gamma^{2}}E\left[M_{\ell ,2}^{\prime }\left(\alpha G+\mu SY;\frac{\tau }{\gamma }\right)\right]+\frac{\tau }{2} & =0. \end{array}$$

Next, we show how these equations simplify to the following system of equations (same as (8):

(A57a) $$\begin{array}{cc} E\left[Y\;S\cdot M_{\ell ,1}^{\prime }\left(\alpha G+\mu SY;\lambda \right)\right] & =0, \end{array}$$

(A57b) $$\begin{array}{cc} \lambda^{2}\;\delta \;E\left[\;{\left(M_{\ell ,1}^{\prime }\left(\alpha G+\mu SY;\lambda \right)\right)}^{2}\;\right] & =\alpha^{2}, \end{array}$$

(A57c) $$\begin{array}{cc} \lambda \;\delta \;E\left[G\cdot M_{\ell ,1}^{\prime }\left(\alpha G+\mu SY;\lambda \right)\right] & =\alpha . \end{array}$$

Let $\lambda :=\frac{\tau }{\gamma }$. First, (A57a) is immediate from equation (A56a). Second, substituting γ from (A56c) in (A56d) yields $\tau =\frac{\alpha }{\sqrt{\delta }}$ or $\gamma =\frac{\alpha }{\lambda \sqrt{\delta }}$, which together with (A56b) leads to (A57c). Finally, (A57b) can be obtained by substituting $\gamma =\frac{\alpha }{\lambda \sqrt{\delta }}$ in (A56c) and using the fact that (see Proposition A1):

$$M_{\ell ,2}^{\prime }\left(\alpha G+\mu SY;\lambda \right)=-\frac{1}{2}{\left(M_{\ell ,1}^{\prime }\left(\alpha G+\mu SY;\lambda \right)\right)}^{2}.$$

### Appendix B.5. On the Uniqueness of Solutions to (A57): Proof of Proposition 1

Here, we prove the claim of Proposition 1 through the following lemmas. As discussed in Remark 4, the main part of the proof is showing strict convex-concavity of *F* in (11). Lemma A6 proves that this is the case, and Lemmas A7 and A8 show that this is sufficient for the uniqueness of solutions to (A57). When put together, these complete the proof of Proposition 1.

**Lemma** **A6.** (Strict Convex-Concavity of (A55)). *Let ℓ:R→R be proper and strictly convex function. Further, assume that ℓ is continuously differentiable with $\ell^{\prime }\left(0\right)\neq 0$. In addition, assume that SY has positive density in the real line. Then, the function $F:R^{4}\to R$ defined in (A55) is strictly convex in (α,μ,τ) and strictly concave in γ.*

**Proof.** The claim follows directly from the strict convexity-concavity properties of the expected Moreau-envelope proved in Propositions A5 and A6. Specifically, we apply Proposition A7. □

**Lemma** **A7.** *If the objective function in (A55) is strictly convex in (α,μ,τ) and strictly concave in γ, then (A56) has a unique solution (α,μ,τ,γ).*

**Proof.** Let $(\alpha_{i},\mu_{i},\tau_{i},\gamma_{i}),\;i=1,2,$ be two different saddle points of (A55). For convenience, let $x_{i}:=\left(\alpha_{i},\mu_{i},\tau_{i}\right)$ for i=1,2. By strict-concavity in γ, for fixed values of x:=(α,μ,τ), the value of γ maximizing F(x,γ) is unique. Thus, if $x_{1}=x_{2}$, then it must hold that $\gamma_{1}=\gamma_{2}$, which is a contraction to our assumption of $\left(x_{1},\gamma_{1}\right)\neq \left(x_{2},\gamma_{2}\right)$. Similarly, we can use strict-convexity to derive that $\gamma_{1}\neq \gamma_{2}$. Then, based on the definition of the saddle point and strict convexity-concavity, the following two relations hold for i=1,2:

$$\begin{array}{c} F\left(x_{i},\gamma \right)<F\left(x_{i},\gamma_{i}\right)<F\left(x,\gamma_{i}\right),\;\mathrm{for}\;\mathrm{all}\;x\neq x_{i},\gamma \neq \gamma_{i}. \end{array}$$

We choose $x=x_{2},\gamma =\gamma_{2}$ for i=1 and $x=x_{1},\gamma =\gamma_{1}$ for i=2 to find

$$\begin{array}{c} F\left(x_{1},\gamma_{2}\right)<F\left(x_{1},\gamma_{1}\right)<F\left(x_{2},\gamma_{1}\right),\\ F\left(x_{2},\gamma_{1}\right)<F\left(x_{2},\gamma_{2}\right)<F\left(x_{1},\gamma_{2}\right). \end{array}$$

From the above, it follows that $F\left(x_{1},\gamma_{1}\right)<F\left(x_{2},\gamma_{2}\right)$ and $F\left(x_{1},\gamma_{1}\right)>F\left(x_{2},\gamma_{2}\right)$, which is a contradiction. This completes the proof. □

**Lemma** **A8.** *If (A56) has a unique solution $\left(\alpha^{☆},\mu^{☆},\tau^{☆},\gamma^{☆}\right)$, then (A57) has a unique solution $\left(\alpha^{☆},\mu^{☆},\lambda^{☆}\right)$.*

**Proof.** First, following the same approach of deriving Equations (A57) from (A56) in (A56), it is easy to see that existence of solution $\left(\alpha_{1},\mu_{1},\tau_{1},\gamma_{1}\right)$ to (A57) implies existence of solution $\left(\alpha_{1},\mu_{1},\lambda_{1}:=\frac{\tau_{1}}{\gamma_{1}}\right)$ to (A57). Now, for the sake of contradiction to the statement of the lemma, assume that there are two different triplets $v_{1}:=\left(\alpha_{1},\mu_{1},\lambda_{1}\right)$ and $v_{2}:=\left(\alpha_{2},\mu_{2},\lambda_{2}\right)$ with $\alpha_{1},\alpha_{2},\lambda_{1},\lambda_{2}>0$ and satisfying (Appendix B.4). Then, we can show that both $w_{i}:=\left(\alpha_{i},\mu_{i},\tau_{i},\gamma_{i}\right)\;i=1,2,$ such that:

$$\begin{array}{c} \tau_{i}:=\frac{\alpha_{i}}{\sqrt{\delta }},\;\gamma_{i}=\frac{\alpha_{i}}{\lambda_{i}\sqrt{\delta }},\;\;i=1,2, \end{array}$$

satisfy the system of equations in (A56). However, since $v_{1}\neq v_{2}$, it must be that $w_{1}\neq w_{2}$. This contradicts the assumption of uniqueness of solutions to (A56) and completes the proof. □

## Appendix C. Discussions on the Fundamental Limits for Binary Models

*On the Uniqueness of Solutions to Equation* $\kappa \left(\sigma \right)=\frac{1}{\delta }$

The existence of a solution to the equation $\kappa \left(\sigma \right)=\frac{1}{\delta }$ is proved in the previous section. However, it is not clear if the solution to this equation is unique, i.e., for any δ>1 there exists only one $\sigma_{\mathrm{opt}}>0$ such that $\kappa \left(\sigma_{\mathrm{opt}}\right)=\frac{1}{\delta }.$ If this is the case, then Equation (12) in Theorem 2 can be equivalently written as

$$\sigma_{\mathrm{opt}}=\sigma ,\;s.t.\;\kappa \left(\sigma \right)=\frac{1}{\delta }.$$

Although we do not prove this claim, our numerical experiments in Figure A1 show that κ(·) is a monotonic function for noisy-signed, logistic and Probit measurements, implying the uniqueness of solution to the equation $\kappa \left(\sigma \right)=\frac{1}{\delta }$ for all δ>1.

### Appendix C.1. Distribution of SY in Special Cases

We derive the following densities for SY for the special cases ($∥x_{0}{∥}_{2}=1$):

- *Signed*: $p_{SY}\left(w\right)=\sqrt{\frac{2}{\pi }}\exp\left(-w^{2}/2\right)\;1_{\left\{w\geq 0\right\}}.$
- *Logistic*: $p_{SY}\left(w\right)=\sqrt{\frac{2}{\pi }}\;\frac{\exp(-w^{2}/2)}{1+\exp(-w)}.$
- *Probit*: $p_{SY}\left(w\right)=\sqrt{\frac{2}{\pi }}\;\Phi \left(w\right)\exp\left(-w^{2}/2\right).$

In particular, we numerically observe that for logistic and Probit models; the resulting densities are similar to the density of a gaussian distribution derived according to N(E[SY],Var[SY]). Figure A2 illustrates this similarity for these two models. As discussed in Corollary 1, this similarity results in the tightness of the lower bound achieved for $\sigma_{\mathrm{opt}}$ in Equation (23).

## Appendix D. Proofs and Discussions on the Optimal Loss Function

### Appendix D.1. Proof of Theorem 3

We show that the triplet $\left(\mu =1,\alpha =\sigma_{\mathrm{opt}},\lambda =1\right)$ is a solution to Equations (8) for *ℓ* chosen as in (29). Using Proposition A2 in the Appendix, we rewrite $\ell_{\mathrm{opt}}$ using the Fenchel–Legendre conjugate as follows:

(A58) $$\begin{array}{c} \ell_{\mathrm{opt}}\left(w\right)={\left(q+\alpha_{1}q+\alpha_{2}\log p_{W_{\mathrm{opt}}}\right)}^{☆}\left(w\right)-q\left(w\right), \end{array}$$

where $q\left(w\right)=w^{2}/2$. For a function *f*, its Fenchel–Legendre conjugate is defined as:

$$f^{☆}\left(x\right)=\max_{y}\;xy-f\left(y\right).$$

Next, we use the fact that, for any proper, closed and convex function *f*, it holds that ${\left(f^{☆}\right)}^{☆}=f$ [61] (theorem 12.2). Therefore, noting that $q+\alpha_{1}q+\alpha_{2}\log p_{W_{\mathrm{opt}}}$ is a convex function (see the proof of Lemma A9 in the Appendix), combined with (A58), it yields that

(A59) $$\begin{array}{c} {\left(\ell_{\mathrm{opt}}+q\right)}^{☆}=q+\alpha_{1}q+\alpha_{2}\log p_{W_{\mathrm{opt}}}. \end{array}$$

Additionally, using Proposition A2, we find that $M_{\ell_{\mathrm{opt}}}\left(w;1\right)=q\left(w\right)-{\left(q+\ell_{\mathrm{opt}}\right)}^{☆}\left(w\right),$ which by (A49) reduces to:

$$M_{\ell_{\mathrm{opt}}}\left(w;1\right)=-\alpha_{1}q\left(w\right)-\alpha_{2}\log p_{W_{\mathrm{opt}}}\left(w\right).$$

Thus, by differentiation, we find that $\ell_{\mathrm{opt}}$ satisfies (28) with c=−1, i.e.,

(A60) $$\begin{array}{c} M_{\ell_{\mathrm{opt}},1}^{\prime }\left(w;1\right)=-\alpha_{1}w-\alpha_{2}\cdot \xi_{W_{\mathrm{opt}}}\left(w\right). \end{array}$$

Next, we establish the desired by directly substituting (A60) into the system of equations in (19). First, using the values of $\alpha_{1}$ and $\alpha_{2}$ in (30), as well as the fact that $\kappa (\sigma_{\mathrm{opt}})=1/\delta$, we have the following chain of equations:

(A61) $$\begin{array}{cc} \;E\left[\;{\left(M_{\ell_{\mathrm{opt}},1}^{\prime }\left(W_{\mathrm{opt}};1\right)\right)}^{2}\;\right] & =E\left[{\left(\alpha_{1}W_{\mathrm{opt}}+\alpha_{2}\;\xi_{W_{\mathrm{opt}}}\left(W_{\mathrm{opt}}\right)\right)}^{2}\right]\\ & =\alpha_{1}^{2}\;\left(\sigma_{\mathrm{opt}}^{2}+1\right)+\alpha_{2}^{2}\;I\left(W_{\mathrm{opt}}\right)+2\;\alpha_{1}\alpha_{2}\;E\left[W_{\mathrm{opt}}\cdot \xi_{W_{\mathrm{opt}}}\left(W_{\mathrm{opt}}\right)\right]\\ & =\frac{1+\sigma_{\mathrm{opt}}^{2}\;\left(\sigma_{\mathrm{opt}}^{2}\;I\left(W_{\mathrm{opt}}\right)-1\right)}{\delta^{2}\;\left(\sigma_{\mathrm{opt}}^{2}\;I\left(W_{\mathrm{opt}}\right)+I\left(W_{\mathrm{opt}}\right)-1\right)}=\frac{\sigma_{\mathrm{opt}}^{2}}{\delta^{2}\;\kappa \left(\sigma_{\mathrm{opt}}\right)}\\ \; & =\sigma_{\mathrm{opt}}^{2}/\delta . \end{array}$$

This shows (8b). Second, using again the specified values of $\alpha_{1}$ and $\alpha_{2}$, a similar calculation yields

(A62) $$\begin{array}{cc} \;E\left[M_{\ell_{\mathrm{opt}},1}^{\prime }\left(W_{\mathrm{opt}};1\right)\xi_{W_{\mathrm{opt}}}\left(W_{\mathrm{opt}}\right)\right] & =-E\left[\left(\alpha_{1}W_{\mathrm{opt}}+\alpha_{2}\;\xi_{W_{\mathrm{opt}}}\left(W_{\mathrm{opt}}\right)\right)\;\xi_{W_{\mathrm{opt}}}\left(W_{\mathrm{opt}}\right)\right]\\ \; & =\alpha_{1}-\alpha_{2}\;I\left(W_{\mathrm{opt}}\right)\\ \; & =-1/\delta . \end{array}$$

Recall from (17) that $E\left[G\cdot M_{\ell_{\mathrm{opt}},1}^{\prime }\left(W_{\mathrm{opt}};1\right)\right]=-\sigma_{\mathrm{opt}}\;E\left[M_{\ell_{\mathrm{opt}},1}^{\prime }\left(W_{\mathrm{opt}};1\right)\xi_{W_{\mathrm{opt}}}\left(W_{\mathrm{opt}}\right)\right].$ This combined with (A62) yields (8c). Finally, we use again (A60) and the specified values of $\alpha_{1}$ and $\alpha_{2}$ to find that

(A63) $$\begin{array}{cc} \;E\left[W_{\mathrm{opt}}\cdot M_{\ell_{\mathrm{opt}},1}^{\prime }\left(W_{\mathrm{opt}};1\right)\right] & =E\left[W_{\mathrm{opt}}\cdot \left(-\alpha_{1}W_{\mathrm{opt}}-\alpha_{2}\;\xi_{W_{\mathrm{opt}}}\left(W_{\mathrm{opt}}\right)\right)\right]\\ & =-\alpha_{1}\;E\left[W_{\mathrm{opt}}^{2}\right]-\alpha_{2}\;E\left[W_{\mathrm{opt}}\;\xi_{W_{\mathrm{opt}}}\left(W_{\mathrm{opt}}\right)\right]\\ & =-\alpha_{1}\left(\sigma_{\mathrm{opt}}^{2}+1\right)-\alpha_{2}\int_{-\infty }^{\infty }w\;p_{W_{\mathrm{opt}}}^{\prime }\left(w\right)\;dw\\ & =-\alpha_{1}\left(\sigma_{\mathrm{opt}}^{2}+1\right)+\alpha_{2}\\ & =\sigma_{\mathrm{opt}}^{2}/\delta . \end{array}$$

However, using (17), it holds that

$$\begin{array}{cc} \;E & \left[W_{\mathrm{opt}}\cdot M_{\ell_{\mathrm{opt}},1}^{\prime }\left(W_{\mathrm{opt}};1\right)\right]=\\ & -\sigma_{\mathrm{opt}}^{2}\;E\left[M_{\ell_{\mathrm{opt}},1}^{\prime }\left(W_{\mathrm{opt}};1\right)\xi_{W_{\mathrm{opt}}}\left(W_{\mathrm{opt}}\right)\right]+E\left[Y\;S\cdot M_{\ell_{\mathrm{opt}},1}^{\prime }\left(W_{\mathrm{opt}};\lambda \right)\right]. \end{array}$$

This combined with (A63) and (A62) shows that $E\left[Y\;S\cdot M_{\ell_{\mathrm{opt}},1}^{\prime }\left(W_{\mathrm{opt}};\lambda \right)\right]=0$, as desired to satisfy (8a). This completes the proof of the theorem.

### Appendix D.2. On the Convexity of Optimal Loss Function

Here, we provide a sufficient condition for $\ell_{\mathrm{opt}}\left(w\right)$ to be convex.

**Lemma** **A9.** *The optimal loss function as defined in Theorem 3 is convex if*

$${\left(\log\left(p_{W_{\sigma }}\right)\right)}^{\prime \prime }\left(w\right)\leq -\frac{1}{\sigma^{2}+1},\;\;\;\;\mathrm{for}\;\mathrm{all}\;w\in R\;\mathrm{and}\;\sigma \geq 0.$$

**Proof.** Using (A9) optimal loss function is written in the following form

(A64) $$\begin{array}{c} \ell_{\mathrm{opt}}\left(w\right)={\left(q+\alpha_{1}q+\alpha_{2}\log\left(p_{W_{\mathrm{opt}}}\right)\right)}^{☆}\left(w\right)-q\left(w\right). \end{array}$$

Next, we prove that $q+\alpha_{1}q+\alpha_{2}\log\left(p_{W_{\mathrm{opt}}}\right)$ is a convex function. We first show that both $\alpha_{1}$ and $\alpha_{2}$ are positive numbers for all values of $\sigma_{\mathrm{opt}}$. We first note that, since *G* and SY are independent random variables, $\sigma_{\mathrm{opt}}^{2}I\left(W_{\mathrm{opt}}\right)<\sigma_{\mathrm{opt}}^{2}I\left(\sigma_{\mathrm{opt}}\;G\right)=1$. Therefore,

(A65) $$\begin{array}{c} 1-\sigma_{\mathrm{opt}}^{2}I\left(W_{\mathrm{opt}}\right)>0. \end{array}$$

Additionally, following the Cramer–Rao bound [53] for Fisher information yields that:

$$\begin{array}{cc} I(W_{\mathrm{opt}}) & >\frac{1}{E\left[{\left(W_{\mathrm{opt}}-E\left[W_{\mathrm{opt}}\right]\right)}^{2}\right]}\\ \; & =\frac{1}{1+\sigma_{\mathrm{opt}}^{2}-{\left(E\left[SY\right]\right)}^{2}}. \end{array}$$

Using this inequality for $I(W_{\mathrm{opt}})$, we derive that

(A66) $$\begin{array}{c} \sigma_{\mathrm{opt}}^{2}I\left(W_{\mathrm{opt}}\right)+I\left(W_{\mathrm{opt}}\right)-1>0. \end{array}$$

From (A65) and (A66), it follows that $\alpha_{1},\alpha_{2}>0$. Based on the definition of the random variable $W_{\mathrm{opt}}$:

$$\begin{array}{c} \log p_{W_{\mathrm{opt}}}\left(w\right)=-w^{2}/\left(2\sigma_{\mathrm{opt}}^{2}\right)\;+\log\int_{-\infty }^{\infty }\exp\left(\left(2wz-z^{2}\right)/2\sigma_{\mathrm{opt}}^{2}\right)\;p_{SY}\left(z\right)\;dz+c, \end{array}$$

where *c* is a constant independent of *w*. By differentiating twice, we see that

$$\log\int_{-\infty }^{\infty }\exp\left(\left(2wz-z^{2}\right)/2\sigma_{\mathrm{opt}}^{2}\right)\;p_{SY}\left(z\right)\;dz$$

is a convex function of *w*. Therefore, to prove that $q+\alpha_{1}q+\alpha_{2}\log\left(p_{W_{\mathrm{opt}}}\right)$ is a convex function, it is sufficient to prove that $(1+\alpha_{1}-\alpha_{2}/\sigma_{\mathrm{opt}}^{2})q$ is a convex function or equivalently $1+\alpha_{1}-\alpha_{2}/\sigma_{\mathrm{opt}}^{2}\geq 0$. Replacing values of $\alpha_{1},\alpha_{2}$ and recalling the equation for $\sigma_{\mathrm{opt}}$ yields that

$$1+\alpha_{1}-\alpha_{2}/\sigma_{\mathrm{opt}}^{2}=0,$$

which implies the convexity of $q+\alpha_{1}q+\alpha_{2}\log\left(p_{W_{\mathrm{opt}}}\right)$. To obtain the derivative of $\ell_{opt}$, we use the result in [61] (Cor. 23.5.1), which states that, for a convex function *f*,

$${\left(f^{☆}\right)}^{\prime }={\left(f^{\prime }\right)}^{-1}.$$

Therefore, following (A64),

(A67) $$\begin{array}{c} \ell_{\mathrm{opt}}^{\prime }\left(w\right)={\left(q^{\prime }+\alpha_{1}q^{\prime }+\alpha_{2}{\left(\log\left(p_{W_{\mathrm{opt}}}\right)\right)}^{\prime }\right)}^{-1}\left(w\right)-w. \end{array}$$

Differentiating again and using the properties of inverse function yields that

(A68) $$\begin{array}{c} \ell_{\mathrm{opt}}^{\prime \prime }\left(w\right)=\frac{1}{1+\alpha_{1}+\alpha_{2}{\left(\log\left(p_{W_{\mathrm{opt}}}\right)\right)}^{\prime \prime }\left(g\left(w\right)\right)}-1, \end{array}$$

where

$$g\left(w\right):={\left(q^{\prime }+\alpha_{1}q^{\prime }+\alpha_{2}{\left(\log\left(p_{W_{\mathrm{opt}}}\right)\right)}^{\prime }\right)}^{-1}\left(w\right).$$

Note that the denominator of (A68) is nonnegative since it is second derivative of a convex function. Therefore, it is evident from (A68) that a sufficient condition for the convexity of $\ell_{\mathrm{opt}}$ is that

$$\alpha_{1}+\alpha_{2}{\left(\log\left(p_{W_{\mathrm{opt}}}\right)\right)}^{\prime \prime }\left(w\right)\leq 0,\;\;\;\;\mathrm{for}\;\mathrm{all}\;w\in R,$$

or

$$1-\sigma_{\mathrm{opt}}^{2}I\left(W_{\mathrm{opt}}\right)+{\left(\log\left(p_{W_{\mathrm{opt}}}\right)\right)}^{\prime \prime }\left(w\right)\leq 0.$$

This condition is satisfied if the statement of the lemma holds for $\sigma =\sigma_{\mathrm{opt}}$:

$$\begin{array}{cc} \; & 1-\sigma_{\mathrm{opt}}^{2}I\left(W_{\mathrm{opt}}\right)+{\left(\log\left(p_{W_{\mathrm{opt}}}\right)\right)}^{\prime \prime }\left(w\right)\leq 1-\sigma_{\mathrm{opt}}^{2}I\left(W_{\mathrm{opt}}\right)-\frac{1}{1+\sigma_{\mathrm{opt}}^{2}}<0, \end{array}$$

where we use (A66) in the last inequality. This concludes the proof. □

#### Appendix D.2.1. Provable Convexity of the Optimal Loss Function for Signed Model

In the case of signed model, it can be proved that the conditions of Lemma A9 is satisfied. Since $W_{\sigma }=\sigma G+SY$, we derive the probability density of $W_{\sigma }$ as follows:

$$\begin{array}{c} p_{W_{\sigma }}\left(w\right)=p_{{}_{\sigma G}}\left(w\right)*p_{SY}\left(w\right)=\frac{\exp(-w^{2}/\left(2+2\sigma^{2}\right))}{\sqrt{2\pi (1+\sigma^{2})}}\cdot f\left(w\right), \end{array}$$

where

$$f\left(w\right)=2-2Q(w/\left(\sigma \sqrt{2+2\sigma^{2}}\right)).$$

Direct calculation shows that *f* is a log-concave function for all w∈R. Therefore,

$$\begin{array}{cc} {\left(\log\left(p_{W_{\sigma }}\right)\right)}^{\prime \prime }\left(w\right) & =-\frac{1}{\sigma^{2}+1}+{\left(\log\left(f\right)\right)}^{\prime \prime }\left(w\right)\\ \; & \leq -\frac{1}{\sigma^{2}+1}. \end{array}$$

This proves the convexity of optimal loss function derived according to Theorem 3 when measurements follow the signed model.

## Appendix E. Noisy-Signed Measurement Model

Consider a noisy-signed label function as follows:

$$\begin{array}{c} y_{i}=f_{\varepsilon }\left(a_{i}^{T}x_{0}\right)=\left\{\begin{array}{cc} \mathrm{sign}(a_{i}^{T}x_{0}) & ,w.p.1-\varepsilon ,\\ -\mathrm{sign}(a_{i}^{T}x_{0}) & ,w.p.\varepsilon , \end{array}\right. \end{array}$$

where ε∈[0,1/2].

In the case of signed measurements, i.e., $y_{i}=\mathrm{sign}\left(a_{i}^{T}x_{0}\right)$, it can be observed that for all possible values of δ, the condition (34) in Section 4.2 holds for $x_{s}=x_{0}$. This implies the separability of data and therefore the solution to the optimization problem (3) is unbounded for all δ. However, in the case of noisy signed label function, boundedness or unboundedness of solutions to (3) depends on δ. As discussed in Section 4.2, the minimum value of δ for bounded solutions is derived from the following:

(A69) $$\delta_{f_{\varepsilon }}^{☆}\left(\varepsilon \right):={\left(\min_{c\in R}E\left[{\left(G+c\;S\;Y\right)}_{-}^{2}\right]\right)}^{-1},$$

where $Y=f_{\varepsilon }\left(S\right)$. It can be checked analytically that $\delta_{f_{\varepsilon }}^{☆}$ is a decreasing function of ε with $\delta_{f_{\varepsilon }}^{☆}\left(0^{+}\right)=+\infty$ and $\delta_{f_{\varepsilon }}^{☆}\left(1/2\right)=2$.

In Figure A3, we numerically evaluate the threshold value $\delta_{f_{\varepsilon }}^{☆}$ as a function of the probability of error ε. For $\delta <\delta_{f_{\varepsilon }}^{☆}$, the set of minimizers of the (3) with logistic or hinge loss is unbounded.

The performances of LS, LAD and hinge loss functions for noisy-signed measurement model with ε=0.1 and ε=0.25 are demonstrated in Figure A4a,b, respectively. Comparing performances of least-squares and hinge loss functions suggest that hinge loss is robust to measurement corruptions, as for moderate to large values of δ it outperforms the LS estimator. Theorem 1 opens the way to analytically confirm such conclusions, which is an interesting future direction.

## Appendix F. On LS Performance for Binary Models

### Appendix F.1. Proof of Corollary 2

To get the values of α and μ as in the statement of the corollary, we show how to simplify Equations (8) for $\ell \left(t\right)={\left(t-1\right)}^{2}$. In this case, the proximal operator admits a simple expression:

$${\mathrm{prox}}_{\ell }\left(x;\lambda \right)=\left(x+2\lambda \right)/\left(1+2\lambda \right).$$

In addition, $\ell^{\prime }\left(t\right)=2\left(t-1\right)$. Substituting these in (14a) gives the formula for μ as follows:

$$\begin{array}{cc} 0 & =E\left[YS(\alpha G+\mu SY-1)\right]=\mu \;E\left[S^{2}\right]-E\left[YS\right]\\ & \;\;\;⟹\mu =E[YS], \end{array}$$

where we have also used from (7) that $E[S^{2}]=1$ and *G* is independent of *S*. In addition, since $\ell^{\prime \prime }\left(t\right)=2$, direct application of (A7) gives

$$\begin{array}{c} 1=\lambda \delta \;\frac{2}{1+2\lambda }⟹\lambda =\frac{1}{2(\delta -1)}. \end{array}$$

Finally, substituting the value of λ into (14b), we obtain the desired value for α as follows:

$$\begin{array}{cc} \alpha^{2} & =4\lambda^{2}\delta \;E\left[{\left({\mathrm{prox}}_{\ell }\left(\alpha G+\mu SY;\lambda \right)-1\right)}^{2}\right]\\ \; & =\frac{4\lambda^{2}}{{\left(1+2\lambda \right)}^{2}}\delta \;E\left[{\left(\alpha G+\mu SY-1\right)}^{2}\right]\\ \; & =\frac{4\lambda^{2}\delta }{{\left(1+2\lambda \right)}^{2}}\left(\alpha^{2}+\mu^{2}+1-2\mu E\left[SY\right]\right)\\ \; & =\frac{1}{\delta }\left(\alpha^{2}+1-{\left(E[SY]\right)}^{2}\right)\;\\ \; & ⟹\alpha =\sqrt{1-{\left(E[SY]\right)}^{2}}\cdot \sqrt{\frac{1}{\delta -1}}. \end{array}$$

### Appendix F.2. Discussion

#### Linear vs. Binary

On the one hand, Corollary 2 shows that least-squares performance for binary measurements satisfies

(A70) $$\lim_{n\to \infty }∥\hat{x}-\frac{\mu }{∥x_{0}{∥}_{2}}\cdot x_{0}{∥}_{2}^{2}=\tau^{2}\cdot \frac{1}{\delta -1},$$

where μ is as in (32) and $\tau^{2}:=1-{\left(E\left[SY\right]\right)}^{2}$. On the other hand, it is well-known (e.g., see references in [15] (Sec. 5.1)) that least-squares for (scaled) linear measurements with additive Gaussian noise (i.e., $y_{i}=\rho a_{i}^{T}x_{0}+\sigma z_{i}$, $z_{i}∼N\left(0,1\right)$) leads to an estimator that satisfies

(A71) $$\begin{array}{c} \lim_{n\to \infty }{∥\hat{x}-\rho \cdot x_{0}∥}_{2}^{2}=\sigma^{2}\cdot \frac{1}{\delta -1}. \end{array}$$

Direct comparison of (A70) to (A71) suggests that least-squares with binary measurements performs the same as if measurements were linear with scaling factor $\rho =\mu /∥x_{0}{∥}_{2}$ and noise variance $\sigma^{2}=\tau^{2}=\alpha^{2}\left(\delta -1\right)$. This worth-mentioning conclusion is not new, as it is proved in [40,43,56,62]. We include a short discussion on the relation to this prior work in the following paragraph. We highlight that all these existing results are limited to a least-squares loss unlike our general analysis.

**Prior work.** There is a lot of recent work on the use of least-squares-type estimators for recovering signals from nonlinear measurements of the form $y_{i}=h\left(a_{i}^{T}x_{0}\right)$ with Gaussian vectors $a_{i}$. The original work that suggests least-squares as a reasonable estimator in this setting is due to Brillinger [56]. In his 1982 paper, Brillinger studied the problem in the classical statistics regime (namely, *n* is fixed not scaling with m→+∞) and he proved for the least-squares solution satisfies

$$\lim_{m\to +\infty }\frac{1}{m}{∥\hat{x}-\frac{\mu }{∥x_{0}{∥}_{2}}\cdot x_{0}∥}_{2}^{2}=\tau^{2},$$

where

(A72) $$\begin{array}{cc} \mu & =E[SY],\;\;\;S∼N(0,1),\\ \tau^{2} & =E[{\left(Y-\mu S\right)}^{2}]. \end{array}$$

and the expectations are with respect to *S* and possible randomness of *f*. Evaluating (A72) for $Y=f_{\varepsilon }\left(S\right)$ leads to the same values for μ and $\tau^{2}$ in (A70). In other works, (A70) for δ→+∞ indeed recovers Brillinger’s result. The extension of Brillinger’s original work to the high-dimensional setting (both m,n large) was first studied by Plan and Vershynin [40], who derived (non-sharp) non-asymptotic upper bounds on the performance of constrained least-squares (such as the Lasso). Shortly after, Thrampoulidis et al. [43] extended this result to *sharp* asymtpotic predictions and to regularized least-squares. In particular, Corollary 2 is a special case of the main theorem in [43]. Several other interesting extensions of the result by Plan and Vershynin have recently appeared in the literature (e.g., [41,62,63,64]). However, the one in [43] is the only one to give results that are sharp in the flavor of this paper. Our work, extends the result of Thrampoulidis et al. [43] to general loss functions beyond least-squares. The techniques of Thrampoulidis et al. [43] that have guided the use of the CGMT in our context were also recently applied by Dhifallah et al. [60] in the context of phase-retrieval.

## Appendix G. Fundamental Limits for Gaussian-Mixture Models: Proofs for Section 5

### Appendix G.1. Proof of Corollary 3

The proof follows directly by noting that, when $\ell \left(t\right)={\left(t-1\right)}^{2}$, it holds that $M_{\ell }\left(x;\lambda \right)=\frac{{\left(x-1\right)}^{2}}{2\lambda +1}.$ By inserting this into (38a) and simplifying the equations, we find the value of μ:

$$\mu =\frac{r}{1+r^{2}}.$$

Similarly, we derive λ using Equation (38c):

$$\lambda =\frac{1}{2(\delta -1)}.$$

Substituting these values of μ and λ into (38b) yields that

$$\alpha^{2}=\frac{1}{\delta -1}\cdot \frac{1}{r^{2}+1}\;.$$

Recalling that $\sigma_{LS}=\alpha /\mu$ concludes the proof.

### Appendix G.2. Proof of Theorem 5

The high-level steps of the proof follow the proof of Theorem 2. First, we note that by scaling the loss function *ℓ* the value of $\sigma_{\ell }$ does not change. In particular, if $\tilde{\ell }\left(t\right):=C_{1}\ell \left(C_{2}t\right)$ for arbitrary constants $C_{1}>0,C_{2}\neq 0$, it is not hard to see that ${\hat{x}}_{\tilde{\ell }}=1/C_{2}\;{\hat{x}}_{\ell }$ is the minimizer of (3). Thus, we conclude from (40) that $\sigma_{\tilde{\ell }}=\sigma_{\ell }$. With this observation, consider the function $\tilde{\ell }:R\to R$ such that $\tilde{\ell }\left(t\right)=\frac{\lambda }{\mu^{2}}\ell \left(\mu \;t\right)$. Then, notice that

$$M_{\ell ,1}^{\prime }\left(x;\lambda \right)=\frac{1}{\lambda }M_{\tilde{\ell },1}^{\prime }\left(x/\mu ;1\right).$$

Using this relation in (38) and setting $\sigma :=\sigma_{\ell }=\alpha /\mu$, the system of equations in (38) can be equivalently rewritten in the following convenient form, where $Z_{\sigma }=\sigma W_{1}+W_{2}$:

(A73a) $$\begin{array}{cc} & E\left[W_{2}\cdot M_{\tilde{\ell },1}^{\prime }\left(Z_{\sigma };1\right)\right]=0, \end{array}$$

(A73b) $$\begin{array}{cc} & E\left[\;{\left(M_{\tilde{\ell },1}^{\prime }\left(Z_{\sigma };1\right)\right)}^{2}\;\right]=\sigma^{2}/\delta \;, \end{array}$$

(A73c) $$\begin{array}{cc} & E\left[W_{1}\cdot M_{\tilde{\ell },1}^{\prime }\left(Z_{\sigma };1\right)\right]=\sigma /\delta \;. \end{array}$$

Next, we show how to use (A73) to derive an equivalent system of equations in terms of only $Z_{\sigma }$. Starting with (A73c), we have

(A74) $$E\left[W_{1}\cdot M_{\tilde{\ell },1}^{\prime }\left(Z_{\sigma };1\right)\right]=\frac{1}{\sigma }\;\int \int \;x\;M_{\tilde{\ell },1}^{\prime }\left(x+y;1\right)p_{\sigma W_{1}}\left(x\right)p_{W_{2}}\left(y\right)dxdy,$$

where recall that $p_{\sigma W_{1}}\left(x\right)=\frac{1}{\sigma \sqrt{2\pi }}e^{-\frac{x^{2}}{2\sigma^{2}}}$. Since it holds that $p_{\sigma W_{1}}\left(x\right)=\frac{-\sigma^{2}}{x}p_{\sigma W_{1}}^{\prime }\left(x\right)$, using (A74) yields that

$$\begin{array}{cc} \;E\left[W_{1}\cdot M_{\tilde{\ell },1}^{\prime }\left(Z_{\sigma };1\right)\right] & =-\sigma \;\int \int \;M_{\tilde{\ell },1}^{\prime }\left(x+y;1\right)p_{\sigma W_{1}}^{\prime }\left(x\right)p_{W_{2}}\left(y\right)dxdy\\ & =-\sigma \;\int \int \;M_{\tilde{\ell },1}^{\prime }\left(z;1\right)p_{\sigma W_{1}}^{\prime }\left(x\right)p_{W_{2}}\left(z-x\right)dxdz=-\sigma \int M_{\tilde{\ell },1}^{\prime }\left(z;1\right)p_{Z_{\sigma }}^{\prime }\left(z\right)dz, \end{array}$$

where in the last step we use

$$p_{Z_{\sigma }}^{\prime }\left(w\right)=\int p_{\sigma W_{1}}^{\prime }\left(x\right)p_{W_{2}}\left(z-x\right)\;dx.$$

Therefore,

$$\begin{array}{c} E\left[W_{1}\cdot M_{\tilde{\ell },1}^{\prime }\left(Z_{\sigma };1\right)\right]=-\sigma \;E\left[M_{\tilde{\ell },1}^{\prime }\left(Z_{\sigma };1\right)\xi_{Z_{\sigma }}\left(Z_{\sigma }\right)\right]. \end{array}$$

This combined with (A73c) gives

$$E\left[M_{\tilde{\ell },1}^{\prime }\left(Z_{\sigma };1\right)\xi_{Z_{\sigma }}\left(Z_{\sigma }\right)\right]=-1/\delta .$$

Second, multiplying (A73c) with $\sigma^{2}$ and adding it to (A73a) yields

(A75) $$\begin{array}{cc} E\left[Z_{\sigma }\cdot M_{\tilde{\ell },1}^{\prime }\left(Z_{\sigma };1\right)\right] & =\sigma^{2}/\delta . \end{array}$$

Putting these together, we conclude with the following system of equations which is equivalent to (A73),

(A76a) $$\begin{array}{cc} & E\left[Z_{\sigma }\cdot M_{\tilde{\ell },1}^{\prime }\left(Z_{\sigma };1\right)\right]=\sigma^{2}/\delta , \end{array}$$

(A76b) $$\begin{array}{cc} & E\left[\;{\left(M_{\tilde{\ell },1}^{\prime }\left(Z_{\sigma };1\right)\right)}^{2}\;\right]=\sigma^{2}/\delta , \end{array}$$

(A76c) $$\begin{array}{cc} & E\left[M_{\tilde{\ell },1}^{\prime }\left(Z_{\sigma };1\right)\xi_{Z_{\sigma }}\left(Z_{\sigma }\right)\right]=-1/\delta . \end{array}$$

Next, considering (A76a) and (A76c), the following holds for any $c_{1},c_{2}\in R$,

(A77) $$E\left[\left(c_{1}Z_{\sigma }+c_{2}\;\xi_{Z_{\sigma }}\left(Z_{\sigma }\right)\right)\cdot M_{\tilde{\ell },1}^{\prime }\left(Z_{\sigma };1\right)\right]=c_{1}\sigma^{2}/\delta -c_{2}/\delta .$$

Applying Cauchy–Schwarz inequality to the LHS of (A77) gives

$$\begin{array}{cc} \; & {\left(c_{1}\sigma^{2}/\delta -c_{2}/\delta \right)}^{2}={\left(E\left[\left(c_{1}Z_{\sigma }+c_{2}\;\xi_{Z_{\sigma }}\left(Z_{\sigma }\right)\right)\cdot M_{\tilde{\ell },1}^{\prime }\left(Z_{\sigma };1\right)\right]\right)}^{2} \end{array}$$

(A78) $$\begin{array}{c} \leq E\left[{\left(c_{1}Z_{\sigma }+c_{2}\;\xi_{Z_{\sigma }}\left(Z_{\sigma }\right)\right)}^{2}\right]E\left[{\left(M_{\tilde{\ell },1}^{\prime }\left(Z_{\sigma };1\right)\right)}^{2}\right]. \end{array}$$

By considering (A76b), $E[Z_{\sigma }\xi_{Z_{\sigma }}\left(Z_{\sigma }\right)]=-1$ (follows from integration by parts) and $E\left[{\left(\xi_{Z_{\sigma }}\left(Z_{\sigma }\right)\right)}^{2}\right]=I\left(Z_{\sigma }\right)={\left(\sigma^{2}+1\right)}^{-1}$, we simplify (A78) to the following:

$${\left(c_{1}\sigma^{2}/\delta -c_{2}/\delta \right)}^{2}\leq \left(c_{1}^{2}\left(\sigma^{2}+1+r^{2}\right)+c_{2}^{2}/\left(\sigma^{2}+1\right)-2c_{1}c_{2})\right)\sigma^{2}/\delta .$$

Choosing $c_{1}=1$ and $c_{2}=\left(1+r^{2}\right)\left(1+\sigma^{2}\right)$ and simplifying both sides, we derive the lower bound for $\sigma^{2}$:

$$\sigma^{2}\geq \frac{1+r^{2}}{r^{2}}\cdot \frac{1}{\left(\delta -1\right)}.$$

This completes the proof of theorem.
